# Characterization of small, deeply located soft-tissue tumors: Conventional magnetic resonance imaging features and apparent diffusion coefficient for differentiation between non-malignancy and malignancy

**DOI:** 10.1371/journal.pone.0232622

**Published:** 2020-05-07

**Authors:** Ji Hyun Lee, Hyun Su Kim, Young Cheol Yoon, Sung Wook Seo, Min Jae Cha, Wook Jin, Jang Gyu Cha

**Affiliations:** 1 Department of Radiology, Samsung Medical Center, Sungkyunkwan University School of Medicine, Seoul, Korea; 2 Department of Orthopedic Surgery, Samsung Medical Center, Sungkyunkwan University School of Medicine, Seoul, Korea; 3 Department of Radiology, Chung-Ang University College of Medicine, Chung-Ang University Hospital, Seoul, Korea; 4 Department of Radiology, Kyung Hee University School of Medicine, Kyung Hee University Hospital at Gangdong, Seoul, Korea; 5 Department of Radiology, Soonchunhyang University Bucheon Hospital, Bucheon, Korea; University at Buffalo, UNITED STATES

## Abstract

**Objectives:**

To compare magnetic resonance imaging (MRI) parameters of small, deeply located non-malignant and malignant soft-tissue tumors (STTs).

**Methods:**

Between May 2011 and December 2017, 95 MRIs in 95 patients with pathologically proven STTs of small size (<5 cm) and deep location (66 non-malignant and 29 malignant) were identified. For qualitative parameters, consensus reading was performed by three radiologists for presence of necrosis, infiltration, lobulation, and the tail sign. Apparent diffusion coefficient (ADC) was analyzed by two other radiologists independently. Univariable and multivariable analyses were performed to determine the diagnostic performances of MRI parameters in differentiating non-malignancy and malignancy, and for non-myxoid, non-hemosiderin STTs and myxoid STTs as subgroups. Interobserver agreement for ADC measurement was calculated with the intraclass correlation coefficient.

**Results:**

Interobserver agreement on ADC measurement was almost perfect. On univariable analysis, the malignant group showed a significantly larger size, lower ADC, and higher incidence of all qualitative MRI parameters for all STTs. Size (p = 0.012, odds ratio [OR] 2.57), ADC (p = 0.041, OR 3.85), and the tail sign (p = 0.009, OR 6.47) were independently significant on multivariable analysis. For non-myxoid, non-hemosiderin STTs, age, size, ADC, frequency of infiltration, lobulation, and the tail sign showed significant differences between non-malignancy and malignancy on univariable analysis. Only ADC (p = 0.032, OR 142.86) retained its independence on multivariable analysis. For myxoid STTs, only size and tail sign were significant on univariable analysis without independent significance.

**Conclusions:**

Size, ADC, and incidence of qualitative MRI parameters were significantly different between small, deeply located non-malignant and malignant STTs. Only ADC was independently significant for both overall analysis and the non-myxoid, non-hemosiderin subgroup.

## Introduction

Soft-tissue tumors (STTs) of musculoskeletal regions are a common indication for evaluation by imaging. Characterizing STTs with regard to their histopathologic nature–whether they are benign or malignant–based on imaging studies is crucial in the management of these lesions, and for suggesting the next clinical step, including biopsy. Several reports have recommended biopsy for STTs with a diameter larger than 5 cm, deep location, and interval growth, or when a definitive diagnosis cannot be made with imaging studies; however, currently, there are no established indications for STT biopsy [1–4].

Magnetic resonance imaging (MRI), with its excellent soft-tissue contrast and large field of view, plays a substantial role in assessment of STTs. Several MRI features are helpful in differentiating benign and malignant STTs [5–7]. However, small STTs often possess MRI features that overlap between benign and malignant lesions, and pose a clinical challenge [8–12]. Early diagnosis with proper management of malignant STTs leads to a better prognosis for patients [13], so it is desirable to identify imaging findings that can aid in appropriate radiologic diagnosis of these lesions.

When STTs are superficially located, they tend to be palpable, and changes in their size may be self-monitored by patients. In contrast, deeply located lesions are often not palpable and may even be difficult to sample, especially when these are small or in close proximity to neurovascular bundles [14,15]. Deep location of STTs has been considered to be a risk factor for malignancy, and an indicator of worse prognosis in cases of malignant STTs [5,6,13,16]. However, a significant amount of benign STTs are also deep-seated lesions [5,6,17].

Diffusion-weighted imaging (DWI) allows quantitative assessment of water diffusion in the tissue, expressed as the apparent diffusion coefficient (ADC) value [18]. Increased cellularity leads to restricted water diffusion at microscopic level [19]. With the ability to reflect this property as a numerical value, ADC has been used as a marker for cellularity in tumors of various regions [20,21]. Several recent studies also suggested that DWI may improve diagnostic performance in differentiating benign from malignant STTs [5,19,22].

We hypothesized that ADC value can help to differentiate between non-malignancy and malignancy for small-sized, deeply located STTs, a subset of tumors of clinical importance. We therefore investigated the ability of ADC and conventional MRI parameters to differentiate between non-malignancy and malignancy in these tumors.

## Methods and materials

### Study subjects

The institutional review board approved this study (Samsung Medical Center, IRB File No. 2018-11-070); since this study was retrospective in nature, informed consent was waived. From May 2011 to December 2017, 4013 MRIs on musculoskeletal regions were performed at our institution, including DWI sequences for suspicious soft-tissue or bone tumors. Of these, 594 MRIs with pathologically proven STTs were enrolled. Thereafter, the following exclusion criteria were used: (a) history of previous treatment, such as surgical excision, chemotherapy, or radiation therapy (n = 192); (b) abundant fatty component (e.g. lipoma or well-differentiated liposarcoma) (n = 71); (c) cystic lesion without an enhancing solid portion (n = 35); (d) suboptimal image quality (e.g. severe susceptibility or motion artifact (n = 18); and (e) simple follow-up for the same lesion (n = 17). Lipoma or well-differentiated liposarcoma were excluded considering the diagnostic algorithm for these tumors differs from other STTs (e.g. size, the presence of non-fatty areas, or MDM2 gene amplification) [23].

One radiologist with 3 years’ experience in musculoskeletal MRI (reader I) recorded the location (superficial or deep) of each lesion; tumor location was defined as superficial or deep relative to the superficial investing fascia on axial T2-weighted image. The lesion’s longitudinal, anteroposterior, and transverse dimensions were measured on MRI; the size, defined as the maximum of the three orthogonal dimensions, was recorded. For the purposes of our study, STTs with a deep location and size of less than 5 cm were selected. In total, 95 MRIs in 95 patients were finally included. These patients had a mean age of 46.7 years (range 10–85 years), and included 49 males (mean age 46.8 years, range 10–85 years) and 46 females (mean age 46.6 years, range 18–84 years); 44 of the subjects were overlapped with a previous study [24]. Whereas this previous study developed, validated, and compared nomograms for malignancy prediction in STTs, we compared MRI features of non-malignant and malignant STTs with different inclusion criteria, focusing on small, deeply-located tumors.

### MRI examinations

All examinations were performed using 3.0-T MRI scanners (Intera Achieva or Ingenia, Philips Medical Systems, Best, The Netherlands). Depending on the lesion’s location, various radiofrequency coils and MRI parameters were used. Conventional protocols included axial and coronal turbo spin echo (TSE) T1-weighted imaging (repetition time [TR]/echo time [TE] 400–520 ms/15–16 ms) and axial and sagittal TSE T2-weighted imaging (TR/TE 2,411–5,366 ms/80–100 ms). Axial-plane DWI consisted of 20 transverse slices, and was performed using a single-shot spin-echo echo-planar sequence. Sensitizing diffusion gradients were applied sequentially in the x, y, and z directions (field of view 160–350 mm; matrix size 128 × 128–256 × 256; TR/TE 5,000 ms/61–69 ms; fat suppression, chemical shift-selective; slice thickness 5 mm; echo train length 59–67; number of averages 2; and b-values 0, 400, and 800 s/mm2). An ADC map was generated using all three b-values. After injection of a bolus of gadoterate meglumine (Dotarem^®^, Guerbet, Roissy, France), axial and coronal TSE fat-suppressed T1-weighted imaging (TR/TE 441–561 ms/15–16 ms; fat suppression, chemical shift-selective) was carried out.

### Clinical and imaging parameter analysis

Clinical data, including age, gender, tumor histopathology, anatomic location, and biopsy method (core biopsy or surgical excision) were gathered, based on review of electronic medical records. Cases were categorized as non-malignant or malignant according to the histopathological results; lesions with intermediate biologic potential were deemed non-malignant [25]. For non-malignant lesions confirmed by core biopsy, the follow-up period of imaging was also recorded.

All MRI analyses were performed using a picture-archiving and communication system (Centricity RA1000 Workstation, GE Healthcare, Chicago, IL, USA). Conventional image parameters included size and qualitative parameters. The following qualitative parameters were analyzed by three radiologists (with 20, 18, and 13 years’ experience in musculoskeletal radiology) to achieve consensus; they were blinded to the clinical information and histopathological results. Infiltration was considered to be present in lesions with indistinct margins. Lobulation was considered present when two or more projections were noted at the margin. Necrosis was deemed present if a fluid-like signal with an irregular margin was observed with no necrotic fluid contrast enhancement. The tail sign was considered present when linear enhancement along the aponeurosis extended from tumor margins [10,26].

Another two radiologists (readers I and II, with 3 and 5 years’ experience in musculoskeletal MRI, respectively) who were blinded to the clinical information and histopathologic results, evaluated the DWI parameters and measured mean ADC values independently. For each lesion, one axial plane was selected that showed the largest tumor section diameter. With conventional images used for reference, regions of interest were manually drawn onto the ADC map maximally within the contrast-enhancing area [5,24]. The most peripheral portion of each lesion was excluded, to minimize partial-volume effects. Regions with necrosis, cystic changes, or dense calcification were also avoided.

Myxoid and hemosiderin components of STTs have been reported as sources of inconsistency in the characterization of malignant STTs using DWI [27,28]. We presumed that uneven distribution of tumor histology with those components, between the non-malignant and malignant group, may lead to misinterpretation of the diagnostic performance of ADC in differentiating the two. We therefore performed subgroup analysis. STTs were classified into three categories: myxoid; hemosiderin; and non-myxoid, non-hemosiderin [5]. An STT was classified as myxoid if it had an obvious myxoid component on the pathologic report, and if it showed a fluid-like, high-signal intensity region on the T2-weighted image, with heterogeneous enhancement. An STT was classified as in the hemosiderin group if a hemosiderin deposit was seen on the pathologic report, and if it showed a dark signal intensity region on T2-weighted image. All other tumors were classified into the non-myxoid, non-hemosiderin group, for which subgroup analysis was performed.

### Statistical analysis

Interobserver agreement on the measurement of ADC values between readers I and II was calculated using the intraclass correlation coefficient (ICC). An ICC value of 1.0 was considered to represent perfect agreement; 0.81–0.99, almost perfect agreement; 0.61–0.80, substantial agreement; 0.41–0.60, moderate agreement; 0.21–0.40, fair agreement; and 0.20 or less, slight agreement [29]. Data were represented on Bland-Altman plots. Continuous and categorical variables were summarized as means with standard deviations and frequency (%), respectively. Univariable analysis comparing non-malignant and malignant STTs was performed using the two-sample t-test for continuous variables and chi-squared test or Fisher’s exact test for categorical variables, respectively. Statistically significant imaging variables or variables that were considered relevant were entered into multivariable logistic regression analysis with the Firth correction.

For all MRI parameters and combination of significant features, the area under the curve (AUC) was calculated based on a receiver-operating characteristic curve analysis; differences between AUCs were assessed according to DeLong et al.’s method [30]. The optimal cutoffs for discrimination of non-malignant and malignant STTs were determined by maximizing Youden’s index, and the sensitivity, specificity, and positive and negative predictive values were calculated. The same analyses were performed for the non-myxoid, non-hemosiderin and myxoid subgroups. Differences were considered statistically significant at a *P* value less than 0.05. Statistical analyses were performed using SAS version 9.4 (SAS Institute, Cary, NC), R-3.4.3 (Vienna, Austria; http://www.R-project.org), and MedCalc version 18.11.3 (MedCalc Software bvba, Ostend, Belgium; http://www.medcalc.org; 2019).

## Results

Of the 95 STTs in 95 patients, 66 were non-malignant and 29 were malignant; 48 and 41 were myxoid (40 non-malignant and 8 malignant) and non-myxoid, non-hemosiderin (20 non-malignant and 21 malignant) STTs, respectively. The non-malignant group included 30 male and 36 female patients with a mean age of 45.6 years (range 12–80 years), while the malignant group comprised 19 male and 10 female patients with a mean age of 50.0 years (range 10–85 years). Of the STTs, 29 were located in the thighs, 21 in the arms, 12 in the hands, 12 in the shoulders, 8 in the feet, 7 in the trunks, and 6 in the pelvis. The numbers of non-malignant and malignant STTs diagnosed by core biopsy, surgical excision, and by both were 8 and 3, 33 and 11, and 25 and 15, respectively. Among 8 patients diagnosed with non-malignant STTs on core biopsy, 6 underwent follow-up MRIs, which did not show changes to suggest malignancy (average follow-up period 17 months, range 6–46 months); follow-up MRI was not performed in the other 2 patients. Seven cases of STT with intermediate biologic potential, including fibromatosis (n = 6) and inflammatory myofibroblastic tumor (n = 1), were classified in the non-malignant group. Detailed histopathologic diagnoses of the patients are summarized in Table 1.

**Table 1:** Histopathologic diagnosis of soft tissue tumors.

Tumor classification	Non-malignant STTs (n = 66)	Malignant STTs (n = 29)
**Myxoid STTs (n = 48)**	Schwannoma (n = 31)	
	Intramuscular myxoma (n = 5)	Myxoid liposarcoma (n = 5)
	Benign fibromyxoid tumor (n = 2)	Fibromyxoid sarcoma (n = 2)
	Neurofibroma (n = 1)	Myxofibrosarcoma (n = 1)
	Melanocytic ganglioneuroma (n = 1)	
**STTs with hemosiderin deposition (n = 6)**	Tenosynovial giant cell tumor (n = 6)	N/A
**Non-myxoid, non-hemosiderin STTs (n = 41)**	Fibromatosis (n = 6)	Metastasis (n = 5)
	Nodular fasciitis (n = 5)	Undifferentiated pleomorphic sarcoma (n = 4)
	Hemangioma (n = 3)	Synovial sarcoma (n = 3)
	Vascular leiomyoma (n = 2)	Epithelioid sarcoma (n = 3)
	Benign mesenchymal tumor (*n* = 1)	Spindle-cell sarcoma (n = 2)
	Benign spindle-cell tumor (n = 1)	Alveolar soft part sarcoma (n = 1)
	Inflammatory myofibroblastic tumor (n = 1)	Alveolar rhabdomyosarcoma (n = 1)
	Glomus tumor (n = 1)	Plasma cell myeloma (n = 1)
		Undifferentiated sarcoma (n = 1)

STT, soft tissue tumor; N/A, not applicable.

**Fig 1:**
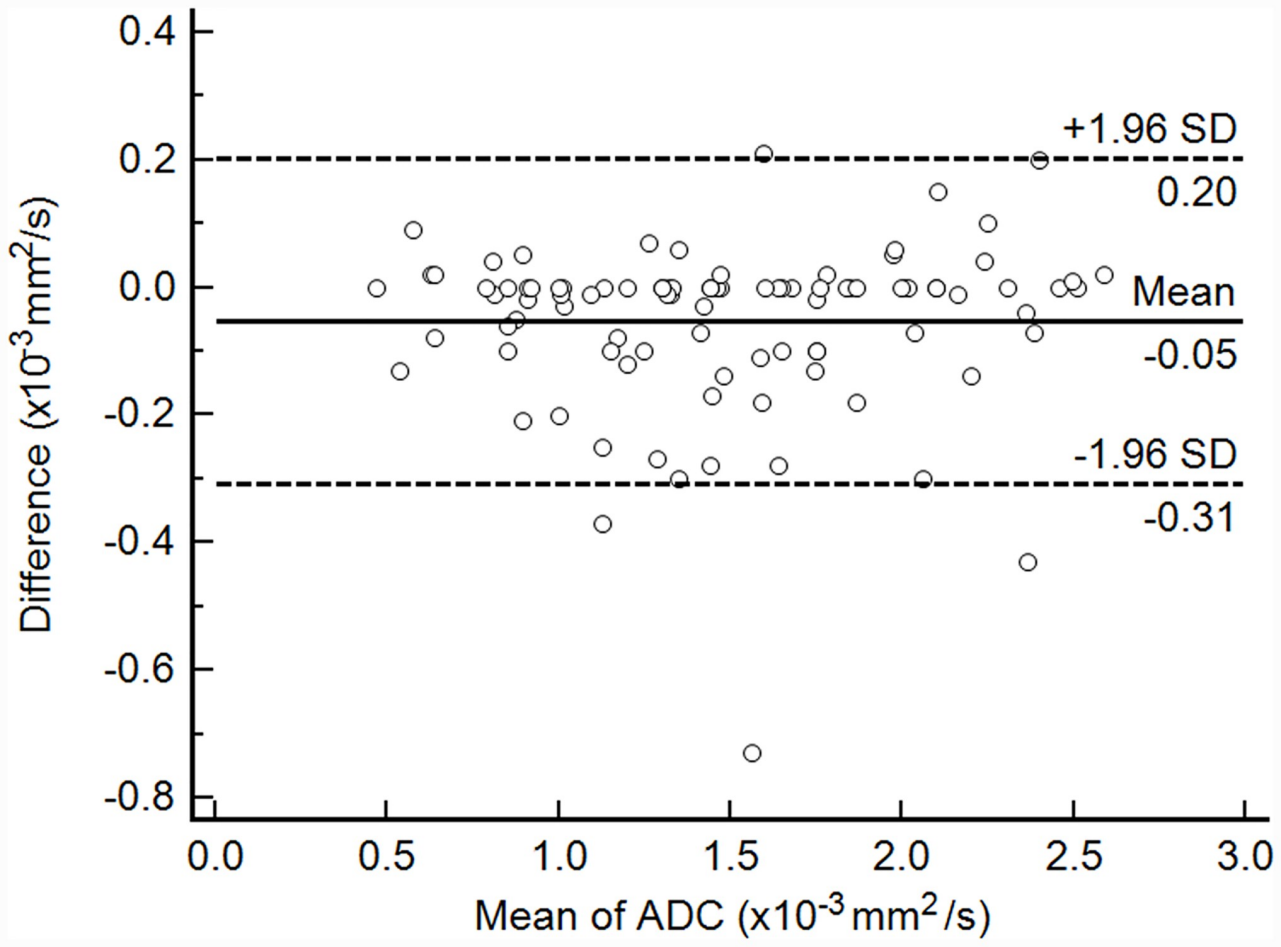
Bland-Altman plot of interobserver agreement for apparent diffusion coefficient ADC values. This plot of ADC measurement data shows the relationship between two readers. Difference (y-axis) between the two readers is plotted against the mean value (x-axis) of their measurements. Solid line, and top and bottom dashed lines indicate mean difference, and upper and lower margins of 95% limits of agreement, respectively. ADC, apparent diffusion coefficient; SD, standard deviation.

**Fig 2:**
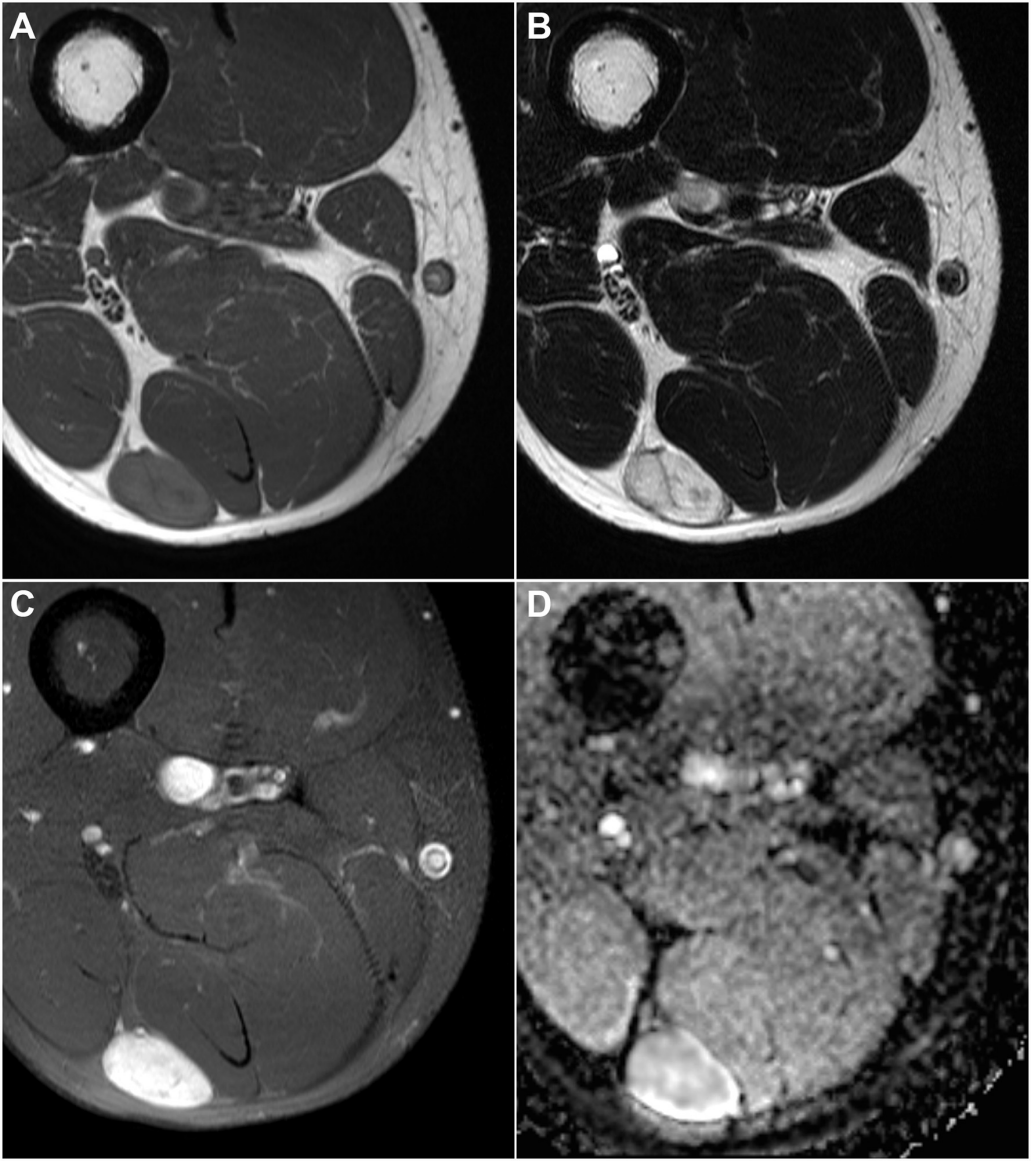
A 44-year-old man with vascular leiomyoma. (A) Axial T1- and (B) T2-weighted images of the right thigh showing a 2.8-cm deeply located oval mass at the intermuscular space of the posterior compartment. (C) Axial fat-suppressed contrast-enhanced T1-weighted image revealed homogeneous strong enhancement. (D) ADC value of the lesion was measured as 1.68 x 10–3 mm2/s. ADC, apparent diffusion coefficient.

**Fig 3:**
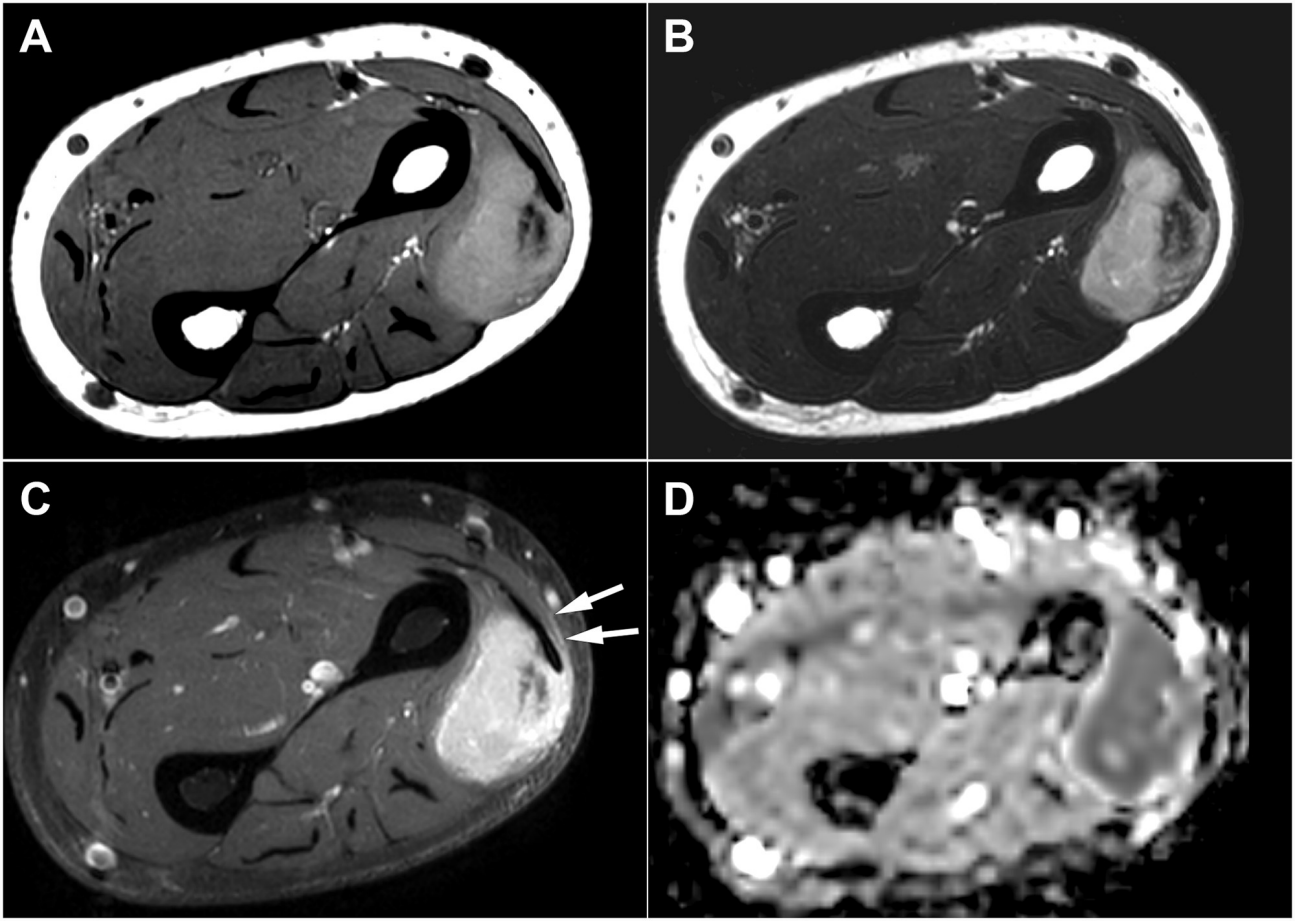
An 80-year-old woman with epithelioid sarcoma. (A) Axial T1- and (B) T2-weighted images of the left forearm showing a 2.7-cm deeply located mass with lobulated contour involving the posterior compartment muscle. (C) Axial fat-suppressed contrast-enhanced T1-weighted image revealed heterogeneous enhancement and the tail sign (arrows). (D) ADC value of the lesion was measured as 1.10 x 10–3 mm2/s. ADC, apparent diffusion coefficient.

There was almost perfect interobserver agreement on measurements of ADC (ICC 0.985, 95% confidence interval (CI) 0.978–0.990), and data obtained by one of the readers were used for comparison (Fig 1). The statistical significance of clinical and imaging parameters for all STTs, myxoid STTs, and non-myxoid, non-hemosiderin STTs on univariable and multivariable analyses are summarized in Table 2. For all STTs, malignant STTs showed significantly larger size, lower ADC, higher frequency of infiltration, lobulation, necrosis, and tail sign; size, ADC, and tail sign retained independent significance on multivariable analysis. In the non-myxoid, non-hemosiderin group, patients with malignant STTs were significantly older, while their tumors were larger, with lower ADC, higher frequency of infiltration, lobulation, and tail sign (Figs 2 and 3). Although necrosis had borderline significance (p = 0.067), it was entered into the multivariable logistic regression analysis, according to previous studies [10,12,31]. Multivariable analysis revealed ADC as the only independent parameter for differentiation of the two groups. ADC was the only factor that retained its independence as a discriminator for both all STTs and non-myxoid, non-hemosiderin STTs in differentiating non-malignant and malignant STTs on multivariable analysis. In contrast, only size and tail sign were significant on univariable analysis in the myxoid group; patients with malignant STTs had larger tumors with higher frequency of tail sign. None of them retained independent significance on multivariable analysis using the same parameters as all STT and non-myxoid non-hemosiderin groups except for necrosis.

**Table 2:** Comparison of clinical and MRI parameters in differentiating between non-malignant and malignant soft-tissue tumors.

	Univariable analysis			Multivariable analysis	
**All STTs (n = 95)**	Non-malignant STTs (n = 66)	Malignant STTs (n = 29)	p value	OR (95% CI)	p value
** Age (years)**	45.6 ± 15.7	50.0 ± 21.3	0.320^a^	N/A	N/A
** Male (%)**	30 (45.5%)	19 (65.5%)	0.072^b^	N/A	N/A
** Size (cm)**	3.00 ± 0.97	3.78 ± 0.70	<0.001^a^	2.57 (1.23–5.38)	0.012
** ADC (10**^**−3**^ **mm**^**2**^**/s)**	1.62 ± 0.50	1.13 ± 0.47	<0.001^a^	3.85 (1.05–14.08)	0.041
** Infiltration (%)**	6 (9.1%)	16 (55.2%)	<0.001^b^	2.33 (0.51–10.70)	0.275
** Lobulation (%)**	40 (60.6%)	27 (93.1%)	0.001^b^	2.31 (0.48–11.15)	0.297
** Necrosis (%)**	4 (6.1%)	8 (27.6%)	0.007^c^	1.93 (0.32–11.74)	0.475
** Tail sign (%)**	6 (9.1%)	16 (55.2%)	<0.001^b^	6.47 (1.59–26.28)	0.009
**Non-myxoid non-hemosiderin STTs (n = 41)**	Non-malignant STTs (n = 20)	Malignant STTs (n = 21)	p value	OR (95% CI)	p value
** Age (years)**	36.6 ± 17.5	50.9 ± 22.7	0.030^a^	N/A	N/A
** Male (%)**	10 (50.0%)	15 (71.4%)	0.160^b^	N/A	N/A
** Size (cm)**	2.89 ± 0.90	3.73 ± 0.67	<0.001^a^	1.37 (0.38–4.92)	0.633
** ADC (10**^**−3**^ **mm**^**2**^**/s)**	1.38 ± 0.40	0.94 ± 0.23	<0.001^a^	142.86 (1.54– >999)	0.032
** Infiltration (%)**	4 (20.0%)	14 (66.7%)	0.004^c^	7.34 (0.80–67.66)	0.079
** Lobulation (%)**	16 (80.0%)	21 (100.0%)	0.033^b^	7.57 (0.25–233.88)	0.248
** Necrosis (%)**	2 (10.0%)	8 (38.1%)	0.067^c^	2.51 (0.19–33.84)	0.488
** Tail sign (%)**	6 (30.0%)	13 (61.9%)	0.043^b^	4.39 (0.50–38.37)	0.181
**Myxoid STTs (n = 48)**	Non-malignant STTs (n = 40)	Malignant STTs (n = 8)	p value	OR (95% CI)	p value
** Age (years)**	50.6 ± 13.0	47.9 ± 18.2	0.618^a^	N/A	N/A
** Male (%)**	18 (45.0%)	4 (50.0%)	1.000^c^	N/A	N/A
** Size (cm)**	3.16 ± 0.96	3.93 ± 0.81	0.040^a^	1.95 (0.69–5.50)	0.208
** ADC (10**^**−3**^ **mm**^**2**^**/s)**	1.85 ± 0.40	1.63 ± 0.58	0.197^a^	5.03 (0.35–71.43)	0.233
** Infiltration (%)**	1 (2.5%)	2 (25.0%)	0.068^c^	0.05 (<0.01–56.52)	0.397
** Lobulation (%)**	19 (47.5%)	6 (75.0%)	0.160^b^	3.87 (0.52–28.83)	0.187
** Necrosis (%)**	2 (5.0%)	0 (0.0%)	1.000^c^	N/A	N/A
** Tail sign (%)**	0 (0.0%)	3 (37.5%)	0.003^c^	58.84 (0.46–>999)	0.100

^a^Determined with the two-sample t-test.

^b^Determined with the chi-square test.

^c^Determined with Fisher’s exact test.

STT, soft tissue tumor; OR, odds ratio; CI, confidence interval; N/A, not applicable; and ADC, apparent diffusion coefficient

**Fig 4:**
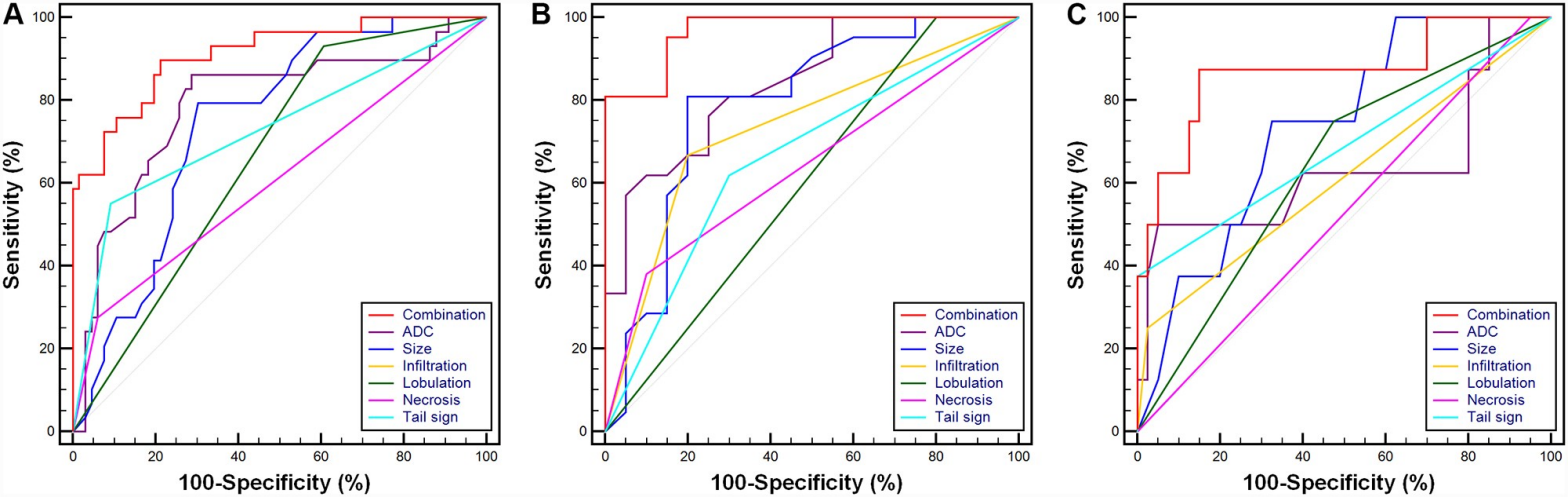
Receiver-operating characteristic analyses of ADC, size, infiltration, lobulation, necrosis, and the tail sign for differentiation of non-malignant and malignant STTs in (A) all STTs, (B) the non-myxoid, non-hemosiderin and (C) myxoid groups. The curves of infiltration and the tail sign were overlapped in the all STTs group. Combinations in (A) all STTs and (B) non-myxoid, non-hemosiderin groups represent size + ADC + infiltration + lobulation + necrosis + tail sign, whereas that in (C) myxoid group represents size + ADC + infiltration + lobulation + tail sign. ADC, apparent diffusion coefficient; STT, soft-tissue tumor.

Overall diagnostic performances of parameters for differentiating non-malignancy and malignancy in all STTs, non-myxoid, non-hemosiderin STTs, and myxoid STTs of small size and deep location are shown in Table 3. Optimal cut-off values of size and ADC for distinguishing non-malignancy and malignancy in all STTs were 3.40 cm and 1.36 x 10–3 mm2/s, respectively; those for non-myxoid, non-hemosiderin STTs were 3.40 cm and 0.91 x 10–3 mm2/s, respectively; those for myxoid STTs were 3.50 cm and 1.32 x 10–3 mm2/s, respectively. Among single parameters, ADC showed the highest AUC in all STTs (0.79, 95% CI 0.68–0.90) and the non-myxoid, non-hemosiderin group (0.84, 95% CI 0.73–0.96), which were significantly higher than those of lobulation (p = 0.017 and p = 0.003 for all STTs and the non-myxoid, non-hemosiderin group, respectively) and necrosis (p = 0.005 and p = 0.030 for all STTs and the non-myxoid, non-hemosiderin group, respectively) (Fig 4). However, differences between AUCs of ADC and other parameters, besides lobulation and necrosis, were not significant. Combination of significant parameters showed the highest AUCs in all STTs (0.91, 95% CI 0.83–0.96) and the non-myxoid, non-hemosiderin group (0.97, 95% CI 0.86–1.00) that were significantly higher than other parameters. In the myxoid group, combination of significant parameters showed higher AUC than those of infiltration (p = 0.010), lobulation (p = 0.007), and necrosis (p < 0.001) (Table 4).

**Table 3:** Diagnostic performance for differentiating between non-malignant and malignant soft tissue tumors.

	AUC	Sensitivity (%)	Specificity (%)	PPV (%)	NPV (%)
**All STTs (n = 95)**					
** Size (cm)**^a^	0.74 (0.64–0.84)	79.3 (63.6–95.0)	69.7 (58.3–81.1)	53.5 (38.0–69.0)	88.5 (79.5–97.4)
** ADC (10**^**−3**^ **mm**^**2**^**/s)**^b^	0.79 (0.68–0.90)	86.2 (72.9–99.6)	71.2 (60.0–82.4)	56.8 (41.6–72.1)	92.2 (84.5–99.8)
** Infiltration**	0.73 (0.63–0.83)	55.2 (35.9–74.4)	90.9 (83.8–98.0)	72.7 (52.5–92.9)	82.2 (73.2–91.2)
** Lobulation**	0.66 (0.59–0.74)	93.1 (83.3–100.0)	39.4 (27.3–51.5)	40.3 (28.2–52.4)	92.9 (82.7–100.0)
** Necrosis**	0.61 (0.52–0.70)	27.6 (10.3–44.9)	93.9 (88.0–99.9)	66.7 (35.4–98.0)	74.7 (65.2–84.3)
** Tail sign**	0.73 (0.63–0.83)	55.2 (35.9–74.4)	90.9 (83.8–98.0)	72.7 (52.5–92.9)	82.2 (73.2–91.2)
** Combination**^c^	0.91 (0.83–0.96)	89.7 (72.6–97.8)	78.8 (65.3–86.7)	63.4 (52.2–73.3)	94.4 (85.2–98.0)
**Non-myxoid, non-hemosiderin STTs (n = 41)**					
** Size (cm)**^a^	0.79 (0.64–0.94)	81.0 (62.6–99.3)	80.0 (60.8–99.2)	81.0 (62.6–99.3)	80.0 (60.8–99.2)
** ADC (10**^**−3**^ **mm**^**2**^**/s)**^d^	0.84 (0.73–0.96)	57.1 (34.1–80.2)	95.0 (84.5–100.0)	92.3 (75.6–100.0)	67.9 (49.4–86.3)
** Infiltration**	0.73 (0.60–0.87)	66.7 (44.7–88.7)	80.0 (60.8–99.2)	77.8 (56.5–99.1)	69.6 (49.2–89.9)
** Lobulation**	0.60 (0.51–0.69)	100.0 (83.9–100.0)	20.0 (7.9–39.2)	56.8 (40.0–73.5)	100.0 (N/A)
** Necrosis**	0.65 (0.51–0.77)	38.1 (15.4–60.8)	90.0 (75.6–100.0)	80.0 (49.8–100.0)	58.1 (39.7–76.5)
** Tail sign**	0.66 (0.51–0.81)	61.9 (39.3–84.6)	70.0 (48.0–92.0)	68.4 (45.4–91.4)	63.6 (41.8–85.5)
** Combination**^c^	0.97 (0.86–1.00)	81.0 (58.1–94.6)	100.0 (75.1–99.9)	94.4 (71.3–99.1)	82.6 (66.2–92.0)
**Myxoid STTs (n = 48)**					
** Size (cm)**^e^	0.73 (0.59–0.85)	75.0 (34.9–96.8)	67.5 (50.9–81.4)	31.6 (20.2–45.7)	93.1 (80.0–97.9)
** ADC (10**^**−3**^ **mm**^**2**^**/s)**^f^	0.64 (0.49–0.77)	50.0 (15.7–84.3)	95.0 (83.1–99.4)	80.0 (33.9–96.9)	90.7 (83.0–95.1)
** Infiltration**	0.61 (0.46–0.75)	25.0 (3.2–65.1)	97.5 (86.8–99.9)	66.7 (17.0–95.1)	86.7 (81.3–90.7)
** Lobulation**	0.64 (0.49–0.77)	75.0 (34.9–96.8)	52.5 (36.1–68.5)	24.0 (15.9–34.6)	91.3 (75.3–97.3)
** Necrosis**	0.53 (0.38–0.67)	0.0 (0.0–36.9)	95.0 (83.1–99.4)	0.0 (N/A)	82.6 (81.6–83.6)
** Tail sign**	0.69 (0.54–0.81)	37.5 (8.5–75.5)	100.0 (91.2–100.0)	100.0 (N/A)	88.9 (82.4–93.2)
** Combination**^g^	0.87 (0.74–0.95)	87.5 (47.3–99.7)	85.0 (70.2–94.3)	50.0 (32.7–67.3)	97.1 (84.0–99.5)

^a^Determined using cut-off value of > 3.40 cm.

^b^Determined using cut-off value of ≤ 1.36 x 10–3 mm2/s.

^c^Combination of significant variables, which are size, ADC, infiltration, lobulation, necrosis, and tail sign.

^d^Determined using cut-off value of ≤ 0.91 x 10–3 mm2/s.

^e^Determined using cut-off value of > 3.50 cm.

^f^Determined using cut-off value of ≤ 1.32 x 10–3 mm2/s.

^g^Combination of significant variables, which are size, ADC, infiltration, lobulation, and tail sign.

Numbers in parentheses are 95% confidence intervals.

AUC, area under the curve; PPV, positive predictive value; NPV, negative predictive value; and ADC, apparent diffusion coefficient.

**Table 4:** Comparison of area under the curves between ADC and other parameters.

Parameters	All STTs		Non-myxoid, non-hemosiderin STTs		Myxoid STTs	
	Difference	p value	Difference	p value	Difference	p value
**ADC–Size**	0.05 (-0.11–0.20)	0.569	0.05 (-0.12–0.23)	0.551	0.10 (-0.26–0.46)	0.593
**ADC–Infiltration**	0.06 (-0.06–0.18)	0.344	0.11 (-0.09–0.31)	0.283	0.02 (-0.22–0.26)	0.847
**ADC–Lobulation**	0.13 (0.02–0.23)	0.017	0.24 (0.08–0.41)	0.003	<0.01 (-0.23–0.23)	0.989
**ADC–Necrosis**	0.18 (0.05–0.31)	0.005	0.20 (0.02–0.39)	0.030	0.11 (-0.17–0.39)	0.442
**ADC–Tail sign**	0.06 (-0.07–0.19)	0.383	0.19 (-0.01–0.38)	0.067	0.05 (-0.25–0.36)	0.739
**Combination–ADC**	0.12 (0.04–0.21)	0.005	0.13 (0.01–0.24)	0.027	0.23 (-0.01–0.48)	0.061
**Combination–Size**	0.17 (0.06–0.27)	0.001	0.18 (0.04–0.32)	0.011	0.13 (-0.10–0.37)	0.263
**Combination–Infiltration**	0.18 (0.10–0.27)	<0.001	0.24 (0.10–0.374)	<0.001	0.26 (0.06–0.45)	0.010
**Combination–Lobulation**	0.25 (0.18–0.32)	<0.001	0.37 (0.28–0.46)	<0.001	0.23 (0.06–0.40)	0.007
**Combination–Necrosis**	0.30 (0.21–0.40)	<0.001	0.33 (0.20–0.45)	<0.001	0.34 (0.17–0.52)	<0.001
**Combination–Tail sign**	0.18 (0.09–0.27)	<0.001	0.31 (0.17–0.45)	<0.001	0.18 (0.00–0.37)	0.054

Numbers in parentheses are 95% confidence intervals.

Combinations in (A) all STTs and (B) non-myxoid, non-hemosiderin groups represent size + ADC + infiltration + lobulation + necrosis + tail sign, whereas that in (C) myxoid group represents size + ADC + infiltration + lobulation + tail sign.

STT, soft tissue tumor and ADC, apparent diffusion coefficient.

## Discussion

Although MRI plays an important role in determining the histopathologic nature of STTs, non-malignant and malignant STTs show overlapping MRI features [8–12]. Excluding characteristic non-malignant tumors for which specific diagnosis can be made based on MRI, such as lipomas or cysts, the ability to discriminate further declines for differentiating non-malignant and malignant STTs [6]. The majority of previous studies regarding MRI findings in STTs consistently reported that lesion size was a significant predictor of malignancy [9,31–38]; deep location has also been regarded as an established risk factor for malignancy [31,33,39]. We aimed to investigate MRI features of non-malignant and malignant STTs with small size and deep location, as they are among the most challenging cases for imaging diagnoses, and cannot be reliably evaluated based on patient-reported size change. Although there have been no established size criteria suggesting malignancy, we selected a maximum diameter of 5 cm as the criterion for differentiating small and large-sized STTs, based on previous guidelines and studies [7,38,40].

Our study revealed that malignant STTs of small size and deep location showed significantly lower ADC values, compared with their non-malignant counterparts [41,42]. This was true for analysis performed in all STTs, as well as subgroup analysis performed in the non-myxoid, non-hemosiderin group. Our study agrees with previous literature reporting ADC as a significant parameter with potential to aid in differentiation of non-malignant and malignant STTs [5,21,22,43]. Furthermore, the diagnostic performance of ADC in terms of AUC was the highest among the imaging parameters, for all STTs as well as the non-myxoid, non-hemosiderin group. However, studies with contrasting results have also reported that substantial overlap exists between ADC values for non-malignant and malignant STTs [28,44], possibly owing to histopathologic heterogeneity of STTs. ADC values can be affected by myxoid matrix or hemosiderin within the tumor, which makes radiologic diagnosis based on ADC values alone quite difficult [27,28]. Therefore, we sought to identify diagnostic performance of ADC for non-myxoid, non-hemosiderin STTs as a subgroup analysis. As was the case for all STTs, our result suggested that ADC measurements could be useful in characterization of non-myxoid, non-hemosiderin tumors.

Our study further strengthened the importance of lesion size in distinguishing non-malignant and malignant STTs, even for the small-sized tumors. With a cut-off value of 3.40 cm, size showed fair diagnostic performance, based on AUC for all STTs and non-myxoid, non-hemosiderin STTs of small size and deep location. However, it retained independent significance only in the all STTs group, and not in the non-myxoid, non-hemosiderin STT group on multivariable analysis. This result is partially comparable with those of Song et al. [5], which included STTs of various sizes and reported that size was not a significant discriminator of non-malignancy and malignancy for non-myxoid, non-hemosiderin STTs. While difficult to estimate due to limited number of STTs for each histologic subtype, there may be a difference in relation between size and malignant potential between myxoid and non-myxoid STTs. These results may also stress the importance of ADC value as a potentially key parameter for discriminating non-malignancy and malignancy in non-myxoid, non-hemosiderin STTs.

Although there has been no meta-analysis regarding MRI features distinguishing non-malignant from malignant STTs, several studies reported that infiltration, lobulation, necrosis, and tail sign suggest malignant STTs [10,12,26,31,45]. The frequencies of all four qualitative MRI parameters were higher in malignant STTs than in non-malignant STTs, for all STTs and the non-myxoid, non-hemosiderin group; statistical significance was noted, except for necrosis in the non-myxoid, non-hemosiderin group. On multivariable analysis of all STTs, tail sign was the only qualitative MRI parameter that retained independent significance; no qualitative MRI parameter retained significance on multivariable analysis in the non-myxoid, non-hemosiderin group.

Our study results suggest that qualitative MRI parameters, which were reported to be helpful in differentiating non-malignant and malignant STTs [10,12,26,31,45], can also be helpful discriminators in STTs of small size. However, presence of these features is determined based on subjective analysis, which can suffer from difficulties in judgement and interobserver disagreement, especially if lesions are small. Considering these limitations, comprehensive analysis with qualitative and quantitative MRI parameters would be particularly important in differentiation of non-malignancy and malignancy in small-sized, deeply located STTs.

We acknowledge several limitations of our study. First, the MRIs were analyzed retrospectively, with variable imaging parameters according to the lesion location. Second, the large range in CIs of ADC in the non-myxoid, non-hemosiderin subgroup analysis possibly indicates less precise estimates of underpowered study, which might be explained by small sample size. Third, the use of 0 s/mm2 for the first b-value instead of 50 s/mm2 may have led to a perfusion-related contribution to the ADC measurement [46]. Fourth, there is a possibility of patient selection bias because we excluded lipomas, well-differentiated liposarcomas, and cystic tumors without solid components, and only patients who had histologic confirmation were included in this study. In addition, with small numbers of malignant myxoid or hemosiderin tumors and high proportion of schwannomas in the non-malignant group, it is necessary to interpret our study results with caution. Fifth, interobserver agreement was not evaluated for qualitative parameters due to consensus analyses; size was measured by only one reader, which is another limitation. Sixth, the amount of myxoid component within the myxoid STTs were not quantitatively analyzed, which may have affected analysis results. Finally, whether ADC has additional diagnostic value compared with conventional MRI was not investigated. It would be beneficial to investigate in a prospective manner whether ADC can provide added value to conventional MRI parameters in terms of diagnostic performance with larger number of cases in future research.

## Conclusion

In conclusion, size, ADC, and incidence of qualitative MRI parameters were significantly different between non-malignant and malignant small-sized, deeply located STTs. Although size and qualitative parameters were helpful discriminators, ADC was the only independently significant parameter in differentiating non-malignancy and malignancy, for both overall analysis and subgroup analysis of the non-myxoid, non-hemosiderin group, which may suggest it to be potentially more valuable. Further studies with larger numbers of subjects are needed to confirm our findings.

## Supporting information

S1 Data (XLSX)

S2 Data (PDF)
