# Supplementary material for: Characterization of small, deeply located soft-tissue tumors: Conventional magnetic resonance imaging features and apparent diffusion coefficient for differentiation between non-malignancy and malignancy
Source: PLoS One. 2020 May 7;15(5):e0232622. doi: 10.1371/journal.pone.0232622 (PMC7205250; doi:10.1371/journal.pone.0232622)
Supplement: S2 Data — (PDF) [file pone.0232622.s002.pdf]

www.nature.com/scientificreports

SCIENTIFIC REPORTS

OPEN

Received: 12 November 2018
Accepted: 28 February 2019
Published online: 20 March 2019

Development and Validation of Nomograms for Malignancy Prediction in Soft Tissue Tumors Using Magnetic Resonance Imaging Measurements

Ji Hyun Lee[1], Young Cheol Yoon[1], Wook Jin[2], Jang Gyu Cha[3] & Seonwoo Kim[4]

The objective of this study was to develop, validate, and compare nomograms for malignancy prediction in soft tissue tumors (STTs) using conventional and diffusion-weighted magnetic resonance imaging (MRI) measurements. Between May 2011 and December 2016, 239 MRI examinations from 236 patients with pathologically proven STTs were included retrospectively and assigned randomly to training (n = 100) and validation (n = 139) cohorts. MRI of each lesion was reviewed to assess conventional and diffusion-weighted imaging (DWI) measurements. Multivariate nomograms based on logistic regression analyses were built using conventional measurements with and without DWI measurements. Predictive accuracy was measured using the concordance index (C-index) and calibration plots. Statistical differences between the C-indexes of the two models were analyzed. Models were validated by leave-one-out cross-validation and by using a validation cohort. The mean lesion size, presence of infiltration, edema, and the absence of the split fat sign were significant and independent predictors of malignancy and included in the conventional model. In addition to these measurements, the mean and minimum apparent diffusion coefficient values were included in the DWI model. The DWI model exhibited significantly higher diagnostic performance only in the validation cohort (training cohort, 0.899 vs. 0.886, P = 0.284; validation cohort, 0.791 vs. 0.757, P = 0.020). Calibration plots showed fair agreements between the nomogram predictions and actual observations in both cohorts. In conclusion, nomograms using MRI features as variables can be utilized to predict the malignancy probability in patients with STTs. There was no definite gain in diagnostic accuracy when additional DWI features were used.

Soft tissue sarcomas are rare neoplasms of mesenchymal origin, which often require multimodal treatment[1]; in contrast, benign tumors require less aggressive management. Determining whether a soft tissue tumor (STT) is benign or malignant is the most important step of the treatment algorithm. In clinical practice, magnetic resonance imaging (MRI) is the preferred imaging modality for STT characterization, local staging, and preoperative planning[1,2]. Generally, the major criteria for diagnosing malignant STTs using conventional MRI include a large size, deep location, and heterogeneous signal intensity[2–8]; other criteria were also suggested[2,4,5,9–11]. Additionally, several characteristic MRI features such as the "target sign"[12,13], "split fat sign"[10,13,14], and "tail sign"[4,15] were reported to be helpful in diagnosing specific STT subtypes. However, it is challenging to distinguish benign from malignant STTs using only MRI; most STTs have nonspecific imaging findings and substantial overlap exists between the imaging features of malignant and benign lesions[4,5,9,16,17]. Consequently, the unsatisfactory diagnostic performance of MRI for distinguishing benign from malignant STTs was reported previously[18–20]. Diffusion-weighted imaging (DWI), which measures the random motion of water protons and provides a

[1]Department of Radiology, Samsung Medical Center, Sungkyunkwan University School of Medicine, Seoul, Korea. [2]Department of Radiology, Kyung Hee University Hospital at Gangdong, Kyung Hee University School of Medicine, Seoul, Korea. [3]Department of Radiology, Soonchunhyang University Bucheon Hospital, Bucheon, Korea. [4]Statistics and Data Center, Research Institute for Future Medicine, Samsung Medical Center, Seoul, Korea. Correspondence and requests for materials should be addressed to Y.C.Y. (email: youngcheol.yoon@gmail.com)

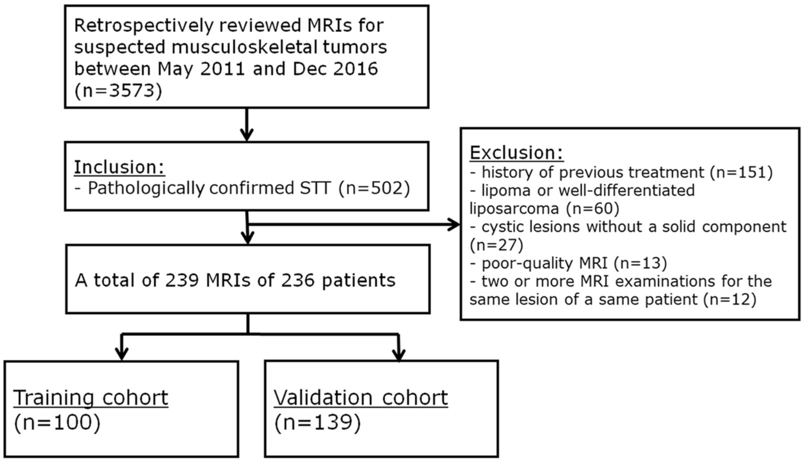

Figure 1. Flow diagram for patient selection. STT, soft tissue tumor.

quantitative parameter of water diffusion in tissue, has been reported to be useful in tumor characterization[21] and treatment response evaluation[22,23] in musculoskeletal imaging. Also, it was suggested that DWI can potentially improve diagnostic performance in the differentiation of benign and malignant STTs[24].

Most previous MRI studies regarding STT differentiation included small numbers of patients, focused on specific subtypes, or focused on a limited number of imaging findings[4,8–10,24]. Few investigations described a systematic imaging approach for differentiating between benign and malignant STTs[8,25].

A nomogram incorporates a variety of factors and is a reliable and pragmatic prediction tool to assess the overall probability of a specific outcome. To the best of our knowledge, no study has used nomograms for STT differentiation. We aimed to build predictive nomograms by combining known clinical and MRI measurements described in the previous literatures[2–12,14,15] and to validate them using a validation cohort. Moreover, the diagnostic performance of a nomogram based on conventional and DWI measurements together was compared with that of a nomogram based on conventional measurements alone to determine whether diagnostic accuracy increases when using DWI measurements.

Materials and Methods

Patients. The institutional review board approved this retrospective study (Samsung Medical Center, 2017-05-087-001); requirement for informed consent was waived. From May 2011 to December 2016, 3,573 MRI examinations including DWI were performed for suspected soft tissue and bone tumors; 502 examinations with pathologically proven STTs were included. Exclusion criteria were (a) previous treatment such as excision, chemotherapy, or radiotherapy (n = 151); (b) lipoma or well-differentiated liposarcoma (n = 60); (c) cystic lesions without a solid component (n = 27); (d) poor-quality MRI (e.g. image distortion with susceptibility artifacts, n = 13); and (e) two or more MRI examinations for the same lesion of a same patient (e.g. simple follow-up MRI, n = 12). A total of 239 MRI examinations from 236 patients (mean age, 48.8 years; range, 9–93 years; 126 male [mean age, 50.6 years; range, 9–90 years] and 110 female patients [mean age, 46.8 years; range, 9–93 years]) were included; 40 of the subjects were overlapped with a previous study[26]; This prior article dealt with tumor spatial heterogeneity whereas in this manuscript we report on predictive nomograms for STT. The numbers of STTs that were pathologically confirmed by image-guided biopsy, surgical excision, and both were 28, 93, and 118, respectively. Patients were assigned randomly to the training (n = 100) or validation (n = 139) cohort (Fig. 1).

MRI techniques. All examinations were performed using 3.0-T MRI scanners (Intera Achieva or Ingenia, Philips Medical Systems, Best, The Netherlands). Depending on the lesion's location, dedicated coils were used with various MRI parameters. Conventional protocols included axial and coronal turbo spin echo (TSE) T_1-weighted imaging (repetition time/echo time, 400–520 ms/15–16 ms) and axial and sagittal TSE T_2-weighted imaging (2,411–5,366 ms/80–100 ms). Axial and coronal TSE fat-suppressed (FS) T_1-weighted imaging (441–561 ms/15–16 ms; fat suppression, chemical shift-selective) was obtained after contrast administration (Gd-DOTA, Dotarem®, Guerbet, Roissy CdG Cedex, France).

Axial-plane DWI was performed using a single-shot spin-echo echo-planar sequence. Sensitizing diffusion gradients were applied sequentially in the x, y, and z directions (field-of-view, 160–350 mm; matrix size, 128 × 128–256 × 256; repetition time/echo time, 5,000 ms/61–69 ms; fat suppression, chemical shift-selective; slice thickness, 5 mm; echo train length, 59–67; number of averages, 2; b-values, 0, 400, and 800 s/mm^2)[24,27,28]. The apparent diffusion coefficient (ADC) map was generated using all b-values. Parallel acquisition was performed with sensitivity-encoding technique (SENSE) by using parallel reduction factor of 1–2 for conventional sequences and 3 for DWI, respectively.

Characteristics	Entire cohort (n = 239)	Training cohort (n = 100)	Validation cohort (n = 139)	P
Age, median (range), years	49 (9–93)	51 (9–87)	49 (9–93)	0.882†
Male (%)	128 (54%)	51 (51%)	77 (55%)	0.502‡
Malignancy (%)	117 (49%)	50 (50%)	67 (48%)	0.784‡
Size				
$Size_{mean}$, median (range), cm	3.5 (0.7–15.8)	3.4 (0.7–13.9)	3.5 (1.0–15.8)	0.892§
$Size_{max}$, median (range), cm	4.4 (1.0–25.0)	4.0 (1.0–24.2)	4.7 (1.0–25.0)	0.665§
Morphology				
Infiltration (%)	92 (38%)	38 (38%)	54 (39%)	0.891‡
Lobulation (%)	189 (79%)	76 (76%)	113 (81%)	0.321‡
Component				
Fat (%)	16 (7%)	6 (6%)	10 (7%)	0.726‡
Fibrosis (%)	26 (11%)	11 (11%)	15 (11%)	0.959‡
Necrosis (%)	43 (18%)	20 (20%)	23 (17%)	0.493‡
Hemorrhage (%)	30 (13%)	14 (14%)	16 (12%)	0.567‡
Septation (%)	56 (23%)	18 (18%)	38 (27%)	0.093‡
Target sign (%)	25 (10%)	12 (12%)	13 (9%)	0.509‡
T_1 heterogeneity (%)				
0	77 (32%)	32 (32%)	45 (32%)	0.680‡
1	77 (32%)	30 (30%)	47 (34%)	
2	43 (18%)	17 (17%)	26 (19%)	
3	42 (18%)	21 (21%)	21 (15%)	
T_2 heterogeneity (%)				
0	12 (5%)	7 (7%)	5 (5%)	0.238‡
1	37 (15%)	14 (14%)	23 (17%)	
2	63 (26%)	21 (21%)	42 (30%)	
3	127 (53%)	58 (58%)	69 (50%)	
Perilesional				
Edema (%)	90 (38%)	34 (34%)	56 (40%)	0.322‡
Split fat sign (%)	34 (14%)	15 (15%)	19 (14%)	0.771‡
Tail sign (%)	72 (30%)	29 (29%)	43 (31%)	0.748‡
Others				
Deep location (%)	178 (74%)	76 (76%)	102 (73%)	0.647‡
NVB invasion (%)	46 (19%)	17 (17%)	29 (21%)	0.455‡
Bone invasion (%)	26 (11%)	10 (10%)	16 (12%)	0.711‡
DWI measurements				
ADC_{mean}, median (range)¶	1.38 (0.47–2.68)	1.39 (0.47–2.68)	1.38 (0.50–2.68)	0.877§
ADC_{min}, median (range)¶	0.87 (0.13–2.43)	0.91 (0.13–2.43)	0.84 (0.15–2.20)	0.218§

Table 1. Descriptive characteristics of the study population. †Independent t test. ‡Chi-squared test. §Mann-Whitney test. ¶10^{-3} mm^2/s. NVB, neurovascular bundle; DWI, diffusion-weighted imaging; ADC, apparent diffusion coefficient.

Clinical and imaging measurement analysis. For clinical data (age, sex, and pathological STT results), electronic medical records were reviewed. Cases were categorized as benign, intermediate or malignant according to the pathological results; Lesions with benign and intermediate biologic behavior were regarded as one category and defined as non-malignant tumors.

Qualitative assessments of conventional image measurements were performed by three radiologists (20, 18, and 13 years of experience in musculoskeletal radiology). The following characteristics were analyzed in consensus by the three radiologists, who were blinded to the clinical information and histopathologic results: Morphology (infiltration, lobulation), component (fat, fibrosis, necrosis, hemorrhage, septation, target sign), T_1 and T_2 heterogeneity, perilesional findings (edema, split fat sign, tail sign), and others (deep location involvement, neurovascular bundle invasion, bone invasion) (Supplemental Material).

Another radiologist (3 years of experience in musculoskeletal MRI) blinded to clinical information and histopathologic results evaluated quantitative MRI measurements: the mean ($size_{mean}$) and maximum ($size_{max}$) sizes and mean (ADC_{mean}) and minimum (ADC_{min}) ADC values. The lesion's longitudinal, anteroposterior, and transverse dimensions were measured on MRI; the maximums and means of the three orthogonal dimensions were recorded. For each lesion, one axial ADC map plane was selected that showed the largest tumor section diameter. The most peripheral portion of each lesion was excluded to minimize partial-volume effects. Referring to axial post-contrast FS T_1-weighted imaging, the region of interest was manually placed on the ADC map maximally within the contrast-enhancing area; regions with necrosis, cystic changes, or dense calcification were avoided.

Training cohort		Validation cohort	
**Non-malignant (n = 50)**	**Malignant (n = 50)**	**Non-malignant (n = 72)**	**Malignant (n = 67)**
Schwannoma (n = 23)	UPS (n = 7)	Schwannoma (n = 26)	Myxoid liposarcoma (n = 12)
Hemangioma (n = 5)	MPNST (n = 5)	Fibromatosis (n = 8)	UPS (n = 8)
Intramuscular myxoma (n = 3)	Myxoid liposarcoma (n = 5)	Neurofibroma (n = 6)	Myxofibrosarcoma (n = 7)
Fibromatosis (n = 3)	DFSP (n = 3)	Nodular fasciitis (n = 4)	Metastatic carcinoma (n = 5)
Neurofibroma (n = 2)	Malignant melanoma (n = 3)	Hemangioma (n = 4)	DFSP (n = 4)
Tenosynovial GCT (n = 2)	Metastatic sarcoma (n = 3)	Tenosynovial GCT (n = 3)	Malignant melanoma (n = 4)
Benign spindle cell tumor (n = 2)	Metastatic carcinoma (n = 3)	Intramuscular myxoma (n = 3)	Synovial sarcoma (n = 4)
Superficial acral fibromyxoma (n = 1)	Alveolar soft part sarcoma (n = 2)	PVNS (n = 2)	Epithelioid sarcoma (n = 3)
Angioleiomyoma (n = 1)	Low-grade fibromyxoid sarcoma (n = 2)	Fibroma (n = 2)	Low-grade fibromyxoid sarcoma (n = 3)
Angiolipoma (n = 1)	ESMC (n = 2)	IMFT (n = 2)	Rhabdomyosarcoma (n = 2)
Cellular angiofibroma (n = 1)	Myxofibrosarcoma (n = 2)	Solitary fibrous tumor (n = 2)	Alveolar soft part sarcoma (n = 2)
Myoepithelioma (n = 1)	Synovial sarcoma (n = 2)	Angioleiomyoma (n = 1)	Lymphoma (n = 2)
Pilomatricoma (n = 1)	Dedifferentiated liposarcoma (n = 1)	Angiomatoid fibrous histiocytoma (n = 1)	MPNST (n = 2)
Spindle cell lipoma (n = 1)	Leiomyosarcoma (n = 1)	Dermatofibroma (n = 1)	Metastatic sarcoma (n = 2)
Vascular leiomyoma (n = 1)	Epithelioid sarcoma (n = 1)	EHE (n = 1)	BPDCN (n = 1)
Benign mesenchymal tumor (n = 1)	Plasma cell myeloma (n = 1)	Intramuscular angioma (n = 1)	ESMC (n = 1)
Benign neurogenic tumor (n = 1)	Ewing sarcoma (n = 1)	Melanocytic ganglioneuroma (n = 1)	Squamous cell carcinoma (n = 1)
	Follicular lymphoma (n = 1)	Chondroid syringoma (n = 1)	Leiomyosarcoma (n = 1)
	Squamous cell carcinoma (n = 1)	Spindle cell lipoma (n = 1)	Pleomorphic sarcoma (n = 1)
	Primary sarcoma (n = 1)	Glomus tumor (n = 1)	Verrucous carcinoma (n = 1)
	Pleomorphic liposarcoma (n = 1)	Vascular leiomyoma (n = 1)	Undifferentiated sarcoma (n = 1)
	Unclassified spindle cell sarcoma (n = 1)		
	Undifferentiated sarcoma (n = 1)		

Table 2. Details of the histopathological diagnoses in the training and validation cohorts. UPS, undifferentiated pleomorphic sarcoma; MPNST, malignant peripheral nerve sheath tumor; DFSP, dermatofibrosarcoma protuberans; ESMC, extraskeletal myxoid chondrosarcoma; GCT, giant cell tumor; PVNS, pigmented villonodular synovitis; IMFT, inflammatory myofibroblastic tumor; EHE, epithelioid hemangioendothelioma; BPDCN, blastic plasmacytoid dendritic cell neoplasm.

Statistical analysis. Continuous and categorical variables are summarized as the median (range) and frequency (%), respectively. For two independent group comparisons, continuous variables and categorical variables were analyzed with the independent t-test or Mann-Whitney test and the chi-squared test or Fisher's exact test, respectively. For use in clinical practice, age, $size_{mean}$, $size_{max}$, ADC_{mean}, and ADC_{min} were categorized using a 5-year scale for age, a 1-cm scale for $size_{mean}$ and $size_{max}$, and 0.1×10^{-3} mm^2/s scale for ADC_{mean} and ADC_{min} to estimate a model predicting malignancy; non-significant cutoffs were excluded. Stepwise selection was applied to the training set using a logistic regression model from all combinations of candidate cutoffs. The model's goodness-of-fit was checked with the R^2 value and Hosmer-Lemeshow test. Likelihood ratio chi-squared statistics for testing the null model against the model with all predictors and the selected models were presented. Variables with $P < 0.05$ were considered independent predictors of malignancy and used for nomogram modeling. Among independent predictors, ADC_{mean} and ADC_{min} were excluded from Model I (the conventional model); they were included in Model II (the DWI model). The nomogram's predictive performance was measured by the concordance index (C-index), which is equivalent to the area under the receiver operating characteristic curve. Models were validated by leave-one-out cross-validation within the training cohort and by using a validation cohort. Then, we selected one model each from Models I and II with the smallest difference between the training and validation cohort C-indexes to determine the most valid model. The nomogram's optimal cutoff for predictive probability was determined by maximizing Youden's index; the sensitivity, specificity, positive predictive value, and negative predictive value were calculated based on the cutoff. The chi-squared test was performed to detect differences in C-indexes between nomograms for Models I and II. In univariate and multivariate analyses, differences were statistically significant at $P < 0.05$.

Nomogram calibrations for both models were assessed for the training and validation cohorts by plotting observed probabilities against nomogram-predicted probabilities of malignancy. Bootstrapping with 1,000 resamples was used to adjust for bias. Statistical analyses were performed using SAS version 9.4 (SAS Institute, Cary, NC, USA) and R version 3.3.2 (R development Core Team, Vienna, Austria).

Characteristics	Non-malignant (n = 50)	Malignant (n = 50)	P
Age			
Age ≤ 50 y	30 (60%)	18 (36%)	0.016†
Age > 50 y	20 (40%)	32 (64%)	
Sex			
Male	26 (52%)	25 (50%)	0.841†
Female	24 (48%)	25 (50%)	
Size			
$Size_{mean}$ ≤ 3 cm	31 (62%)	11 (22%)	<0.001†
$Size_{mean}$ > 3 cm	19 (38%)	39 (78%)	
$Size_{max}$ ≤ 4 cm	34 (68%)	16 (32%)	<0.001†
$Size_{max}$ > 4 cm	16 (32%)	34 (68%)	
Morphology			
Infiltration	6 (12%)	32 (64%)	<0.001‡
Lobulation	31 (62%)	45 (90%)	0.001†
Component			
Fat	2 (4%)	4 (8%)	0.678‡
Fibrosis	3 (6%)	8 (16%)	0.110†
Necrosis	3 (6%)	17 (34%)	<0.001†
Hemorrhage	3 (6%)	11 (22%)	0.021†
Septation	10 (20%)	8 (16%)	0.603†
Target sign	11 (22%)	1 (2%)	0.002†
T_1 heterogeneity			
0	17 (34%)	15 (30%)	0.179†
1	19 (38%)	11 (22%)	
2	6 (12%)	11 (22%)	
3	8 (16%)	13 (26%)	
T_2 heterogeneity			
0	2 (4%)	5 (10%)	0.307‡
1	9 (18%)	5 (10%)	
2	8 (16%)	13 (26%)	
3	31 (62%)	27 (54%)	
Perilesional			
Edema	8 (16%)	26 (52%)	<0.001†
Split fat sign	14 (28%)	1 (2%)	<0.001†
Tail sign	5 (10%)	24 (48%)	<0.001†
Others			
Deep location	37 (74%)	39 (78%)	0.640†
NVB invasion	5 (10%)	12 (24%)	0.062†
Bone invasion	3 (6%)	7 (14%)	0.182†
DWI measurements			
ADC_{mean} < 1.3§	10 (20%)	31 (62%)	<0.001†
ADC_{mean} ≥ 1.3§	40 (80%)	19 (38%)	
ADC_{min} < 0.9§	14 (28%)	36 (72%)	<0.001†
ADC_{min} ≥ 0.9§	36 (72%)	14 (28%)	

Table 3. Demographic and MRI characteristics of non-malignant and malignant soft tissue tumors. NVB, neurovascular bundle; DWI, diffusion-weighted imaging; MRI, magnetic resonance imaging; ADC, apparent diffusion coefficient. †Chi-squared test. ‡Fisher's exact test. §10^{-3} mm^2/s.

Results

Patient demographics. The median age was 51 years (range, 9–87 years) in the training cohort and 49 years (range, 9–93 years) in the validation cohort. Malignant STTs were identified in 50% (50/100 cases) in the training cohort and in 48% (67/139 cases) in the validation cohort. Among 28 STTs that were pathologically confirmed by image-guided biopsy, 15 were benign. 13 of them showed no significant interval change or suspicious features for malignancy during follow up period (average, 15 months; range, 2–46 months); follow up was lost in the other two cases. The pathological reports were different between that from image-guided biopsy and surgical excision in three malignant STTs; false-negative core biopsy results were obtained in two myxoid liposarcomas and a low grade fibromyxoid sarcoma. Lesions with intermediate biologic behavior including fibromatosis,

Variables	Odds ratio (95% CI)	*P*
Model I		
$Size_{mean} > 3$ cm	4.83 (1.61–14.47)	0.005
Infiltration	7.89 (2.49–24.96)	<0.001
Edema	3.95 (1.21–12.94)	0.023
Split fat sign	0.07 (0.01–0.86)	0.038
Model II		
$Size_{mean} > 3$ cm	5.01 (1.56–16.06)	0.007
Infiltration	5.08 (1.51–17.10)	0.009
Edema	2.89 (0.80–10.50)	0.107
Split fat sign	0.05 (<0.01–0.71)	0.027
$ADC_{mean} < 1.3^{\dagger}$	0.33 (0.06–1.86)	0.210
$ADC_{min} < 0.9^{\dagger}$	0.71 (0.14–3.62)	0.677

Table 4. Selected variables used to build the models based on the multivariate analysis. CI, confidence interval; ADC, apparent diffusion coefficient. $^{\dagger}10^{-3}$ mm^2/s.

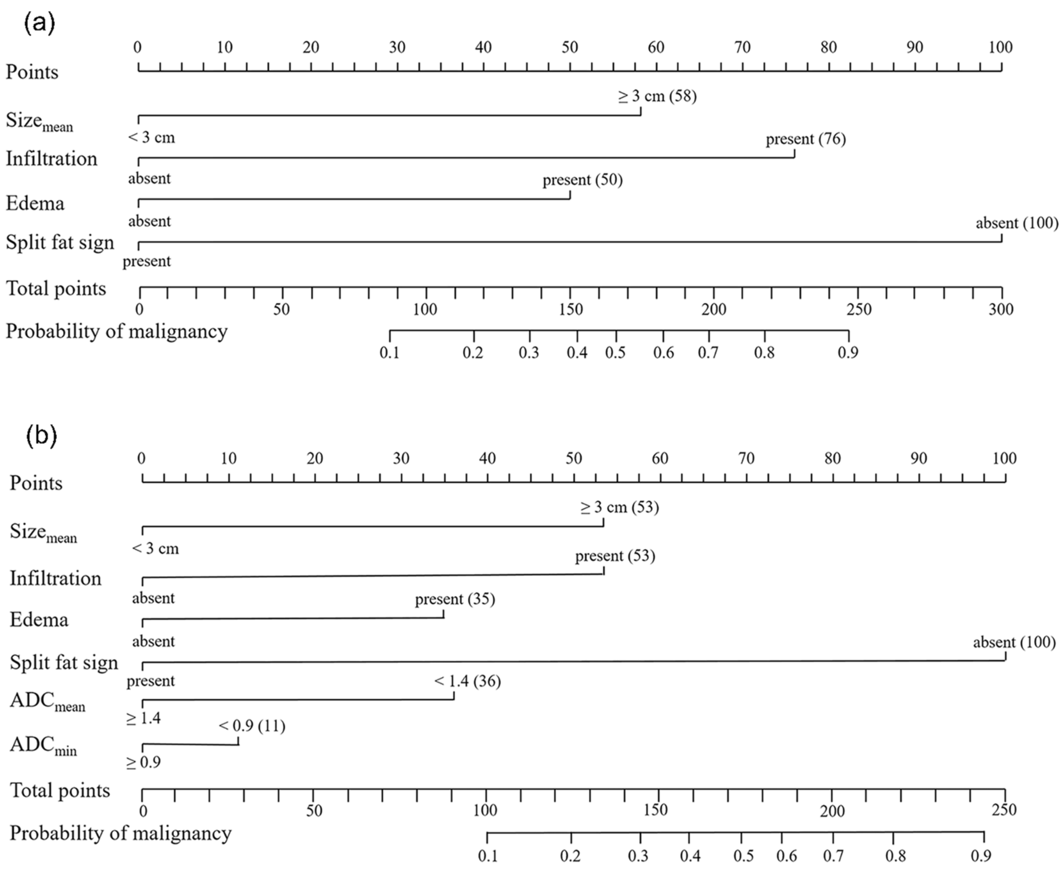

Figure 2. Nomograms for predicting the probability of malignancy in soft tissue tumors by using conventional variables only (**a**: Model I) and by using ADC values in addition to conventional variables (**b**: Model II). ADC, apparent diffusion coefficient.

inflammatory myofibroblastic tumor, epithelioid hemangioendothelioma, solitary fibrous tumor, myoepithelioma, and angiomatoid fibrous histiocytoma were categorized as non-malignant group. Descriptive characteristics for both cohorts are summarized and compared in Table 1. The two cohorts showed similar demographics and MRI features without significant statistical differences. Detailed histopathological diagnoses of the cohorts are summarized in Table 2.

Estimation of the prediction model. The results of the univariate analysis for each candidate cutoff point are shown in Table 3. An age >50 years; $size_{mean} > 3$ cm; $size_{max} > 4$ cm; presence of infiltration, lobulation, necrosis, hemorrhage, edema, or the tail sign; an $ADC_{mean} < 1.3 \times 10^{-3}$ mm^2/s; $ADC_{min} < 0.9 \times 10^{-3}$ mm^2/s; and

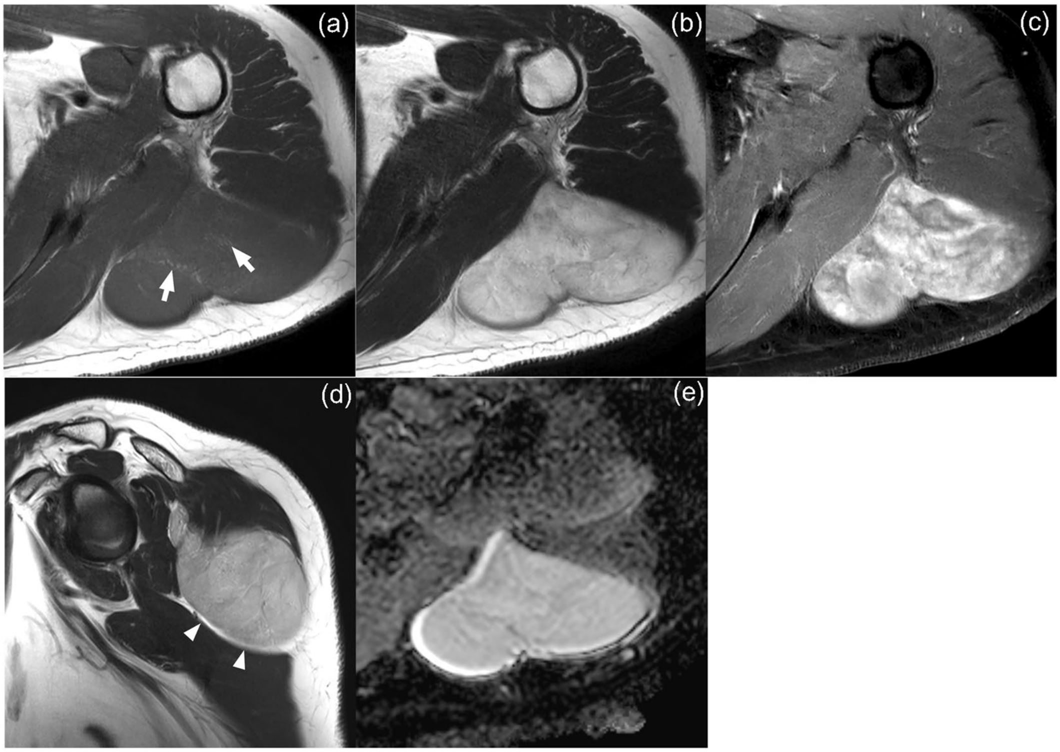

Figure 3. A 53-year-old woman with spindle cell lipoma. (**a**) Axial T1- and (**b**) T2-weighted images of the left shoulder showing a deep-located mass with mean size of 6.4 cm. Scattered areas of high signal intensity on T1-weighted image are noted, suggesting intratumoral fat component (arrows). (**c**) Axial fat-suppressed contrast-enhanced T1-weighted image revealed heterogeneous enhancement. (**d**) Split fat sign was observed between the tumor and triceps brachii muscle on sagittal T2-weighted image (arrowheads). (**e**) The mean and minimum ADC values of the lesion were measured to be 2.60×10^{-3} mm^2/s and 1.90×10^{-3} mm^2/s, respectively. The probability of malignancy was calculated to be less than 0.1 by both models I and II.

absence of the target sign and split fat sign were significant between non-malignant and malignant cases. From all combinations of variables with significant candidate cutoffs and other variables including the absence of the target and split fat signs, a stepwise logistic regression analysis for Model I revealed that a $size_{mean} > 3$ cm, and presence of infiltration, edema, or the split fat sign retained independent significance for predicting malignancy. After the addition of $ADC_{mean} < 1.3 \times 10^{-3}$ mm^2/s and $ADC_{min} < 0.9 \times 10^{-3}$ mm^2/s to these variables, $size_{mean} > 3$ cm, presence of infiltration, and the split fat sign retained independent significance (Table 4). R^2 values were 0.455 and 0.449, and P-values from the Hosmer–Lemeshow test were 0.598 and 0.874 for Models I and II, respectively. Chi-squares from the likelihood ratio for models including all predictors, Model I, and Model II were 70.92, 54.46, and 59.51, respectively.

Construction of nomograms for predicting malignancy. Independent variables for predicting malignancy were used to construct nomograms for Models I and II. The conventional nomogram (Model I) was formulated using conventional variables only, whereas the DWI nomogram (Model II) was formulated using $ADC_{mean} < 1.3 \times 10^{-3}$ mm^2/s and $ADC_{min} < 0.9 \times 10^{-3}$ mm^2/s in addition to conventional variables (Fig. 2). By determining the score from all variables on a total point scale, probabilities of malignancy could be determined by drawing a vertical line to the total score (Figs 3 and 4). In both models, the nomograms showed that the split fat sign contributed most to the probability of malignancy. Other variables showed moderate impacts on the probability of malignancy except for ADC_{min} in Model II. Calibration plots presented fair agreements between the prediction by nomogram and actual observation of malignancy in the training and the validation cohorts (Fig. 5).

Performance of the two models and validation. In the training cohort, sensitivity, specificity, positive predictive value, negative predictive value, and accuracy for Model I were 0.78, 0.86, 0.85, 0.80, and 0.82 using a nomogram cutoff of 176; those for Model II were 0.80, 0.86, 0.85, 0.81, and 0.83 using a nomogram cutoff of 188, respectively. Applying these nomogram cutoffs to the validation cohort, the Model I sensitivity, specificity, positive predictive value, negative predictive value, and accuracy were 0.72, 0.72, 0.71, 0.73, and 0.72; those for Model II were 0.73, 0.76, 0.74, 0.75, and 0.75, respectively. C-index values for Models I and II were 0.886 (95% confidence interval [CI], 0.821–0.951) and 0.899 (95% CI, 0.841–0.958) in the training cohort, respectively; those for Models I and II were 0.757 (95% CI, 0.675–0.839) and 0.791 (95% CI, 0.715–0.867) in the validation cohort, respectively. Cross-validation showed C-index values of 0.853 (95% CI, 0.776–0.930) and 0.878 (95% CI, 0.811–0.946) for

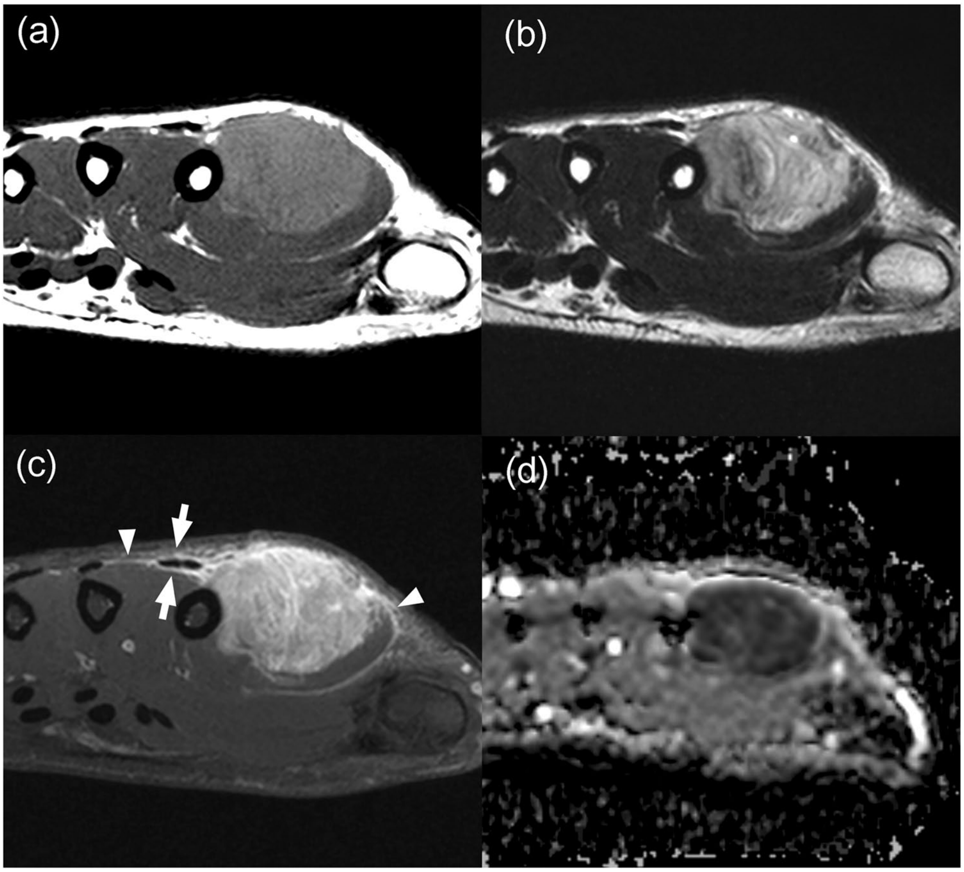

Figure 4. A 20-year-old man with alveolar rhabdomyosarcoma. (**a**) Axial T1- and (**b**) T2-weighted images of the left hand showing a lobulated mass with mean size of 2.9 cm and peritumoral edema (not shown). Split fat sign was not evident. (**c**) Heterogeneous enhancement was seen on the axial fat-suppressed contrast-enhanced T1-weighted image. Infiltration along the extensor tendon (arrows) and tail sign (arrowheads) were noted. (**d**) The mean and minimum ADC values of the lesion were measured to be 0.85×10^{-3} mm^2/s and 0.46×10^{-3} mm^2/s, respectively. The probability of malignancy was calculated to be between 0.8–0.9 by both models I and II.

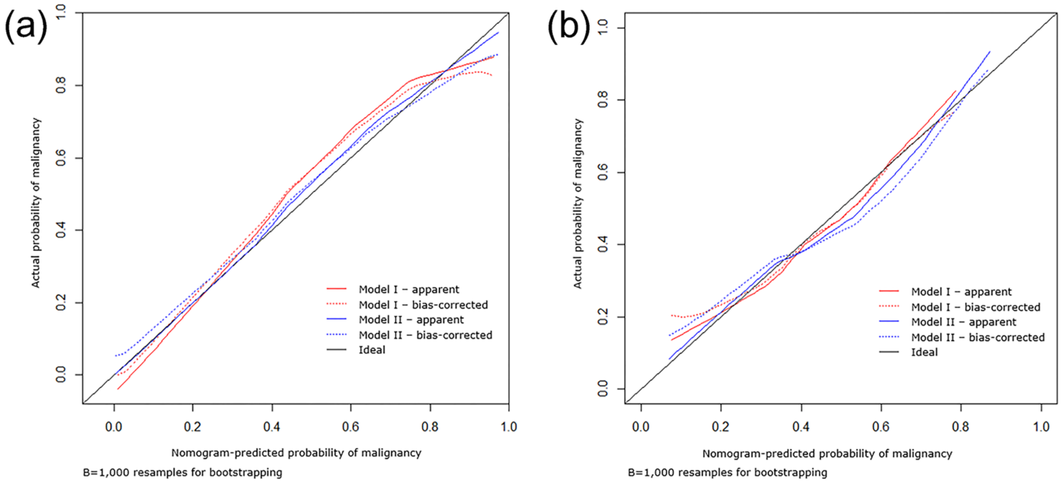

Figure 5. Calibration plots of the probability of malignancy in the (**a**) training and (**b**) validation cohorts. The nomogram-predicted probability of malignancy is plotted on the x-axis; the actual probability of malignancy is plotted on the y-axis. The 45-degree line through the origin represents the perfect calibration model in which the predicted probabilities are identical to the actual probabilities.

Models I and II, respectively. P-values for analyzing C-index differences between the models were 0.284 and 0.020 in the training and validation cohorts, respectively, with statistical significance only in the validation cohort.

Discussion

Despite superior soft tissue contrast and resolution, MRI had limited STT characterization and differential diagnosis ability, with conflicting conclusions reported by previous studies[18–20]. Berquist *et al.*[11] reported a sensitivity of 90–96% and specificity of 82–96% for malignancy prediction using traditional imaging features including size, margins, and signal intensity heterogeneity. However, Kransdorf *et al.*[18] concluded that MRI was incapable of reliably distinguishing between benign and malignant STTs; a specific diagnosis was made in only 24%. Crim *et al.*[20] reported that MRI had insufficient accuracy in differentiating benign from malignant STTs. Considering no single imaging feature was sufficient to distinguish benign from malignant STTs in most previous investigations, we combined individual measurements and formulated nomograms to diagnose STTs simply in daily clinical practice with high diagnostic performance.

Our study demonstrated that the mean size and presence of infiltration, edema, and the split fat sign were independent predictors in differentiating non-malignant and malignant STT. Adding DWI measurements including ADC_{mean} and ADC_{min} improved diagnostic performance significantly only in the validation cohort. Moulton *et al.*[25] reported that lesion size, margination, and edema were the best predictors using a stepwise logistic regression analysis; adding any fourth imaging feature did not improve accuracy. Except for the split fat sign, which they did not evaluate, these findings are comparable to ours.

The split fat sign was described to suggest a slow-growing tumor originating from the intermuscular space around the neurovascular bundle[13]. Although nonspecific, it is a common finding in benign peripheral nerve sheath tumor[13,29]. In our study, of 34 STTs (training and validation cohorts) showing the split fat sign, 30 were non-malignant and four were malignant. Because of high proportions of schwannomas in the non-malignant groups of both the training (23/50, 46%) and validation (26/72, 36%) cohorts, contribution of the split fat sign to the malignancy probability might be overestimated, which is a study limitation. All four malignant STTs showing the split fat sign had slow-growing characteristics[30–32], suggesting that slowly enlarging STTs may demonstrate this sign despite malignancy. Our result is comparable to that of Murphy *et al.*[13], who stated that the split fat sign might be noted in malignant peripheral nerve sheath tumor. They found that fat rims of malignant peripheral nerve sheath tumors were more frequently incomplete because of its aggressive and infiltrative growth pattern, which might improve malignant STT diagnoses showing the split fat sign.

Although some authors found that size was not useful in distinguishing benign from malignant STTs[20], size was consistently a statistically significant predictor of malignancy in most studies[1,3,5–8,33–35]. However, it is unclear from most reports whether they used the maximum or mean tumor diameter. Our results suggested that the mean size was more significant than the maximal size in predicting malignancy, which corresponds with a report by Harish *et al.*[3].

Since Rydholm[6] and Myhre-Jensen[7] described that most malignant STTs are deep whereas only about 1% of all benign STTs are deep, deep location has been regarded as an established risk factor for malignancy[1,2,33]. However, some authors recently reported that depth relationship to fascia is less important as a predictor of malignant potential[8,36], which were also comparable to our results. Considering that the previous literatures by Rydholm[6] and Myhre-Jensen[7] were reported in the early 1980s, we supposed that these contrasting results might have been resulted from advances in diagnostic modality including MRI which helped detecting more deep-seated benign STTs.

We designed and conducted this study hypothesizing that adding DWI measurements could improve accuracy. However, diagnostic accuracy gains were marginal and showed statistical significance between the models only in the validation cohort. These results were partially comparable to those reported by Jeon *et al.*[24]. Although they concluded that adding DWI to conventional MRI can improve the diagnostic performance for the differentiation between malignant and benign STTs, the accuracy were the same for an experienced reader regardless of whether DWI was used or not. Considering their results together with those of our study, we supposed that the added value by using DWI might be limited for experienced readers.

A strength of our study is that we developed systematic imaging approach based on predictive models for overall STT differentiation using nomograms, in contrast to the previous studies which used subjective method[11,17,20] or evaluated only specific subtypes of STTs[3,4,9,10,24]. Further investigations to compare the diagnostic performance of prediction models and conventional non-quantified approach might be necessary.

Our study has several limitations. First, MRI parameters were variable because of the retrospective analysis. Second, our prediction model validity was imperfect with a small difference in diagnostic performance between training and validation cohorts. The C-index values were lower in the validation cohorts, possibly owing to heterogeneous and diverse pathology in the two groups. Additionally, because of randomization when constructing the training and validation cohorts, which is different from true temporal validation, the generalizability of our models might be limited. Nonetheless, we randomized patients to minimize the possibility of bias. All the MRIs were obtained using machines of the same manufacturer, which also could be one of the limitations in terms of generalizability. Third, we excluded lipomas, well-differentiated liposarcomas, and cystic tumors without solid components, which may have resulted in selection bias. Moreover, a high proportion of schwannomas in both cohorts could cause a selection bias, as described above. Fourth, the use of consensus precluded inter-observer variability evaluations. Despite the fact that inter-observer agreement is a significant variable in MRI diagnostic accuracy, we sought to increase confidence for each imaging variable by using three experienced readers' consensus analyses; quantitative measurements were evaluated by only one reader, which is another limitation. Fifth, the use of 0 s/mm^2 for the first b-value instead of 50 s/mm^2 might lead to perfusion related contribution to the ADC measurement[27]. Sixth, STT with intermediate malignancy (e.g., 'locally aggressive' and 'rarely metastasizing') were classified as 'non-malignant' tumors with benign lesions, although they may require specific treatment strategies.

In conclusion, nomograms using MRI features as variables can be utilized to predict the probability of malignancy in patients with STTs. There was no definite gain in diagnostic accuracy for differentiating non-malignant and malignant STTs when additional DWI features were used.

References

1. Group, E. S. E. S. N. W. Soft tissue and visceral sarcomas: ESMO Clinical Practice Guidelines for diagnosis, treatment and follow-up. *Ann Oncol* **25**(Suppl 3), iii102–112, https://doi.org/10.1093/annonc/mdu254 (2014).
2. Jagannathan, J. P., Tirumani, S. H. & Ramaiya, N. H. Imaging in Soft Tissue Sarcomas: Current Updates. *Surg Oncol Clin N Am* **25**, 645–675, https://doi.org/10.1016/j.soc.2016.05.002 (2016).
3. Harish, S., Lee, J. C., Ahmad, M. & Saifuddin, A. Soft tissue masses with "cyst-like" appearance on MR imaging: Distinction of benign and malignant lesions. *Eur Radiol* **16**, 2652–2660, https://doi.org/10.1007/s00330-006-0267-5 (2006).
4. Crombe, A. *et al*. Soft tissue masses with myxoid stroma: Can conventional magnetic resonance imaging differentiate benign from malignant tumors? *Eur J Radiol* **85**, 1875–1882, https://doi.org/10.1016/j.ejrad.2016.08.015 (2016).
5. De Schepper, A. M., Ramon, F. A. & Degryse, H. R. Statistical analysis of MRI parameters predicting malignancy in 141 soft tissue masses. *Rofo* **156**, 587–591, https://doi.org/10.1055/s-2008-1032948 (1992).
6. Rydholm, A. Management of patients with soft-tissue tumors. Strategy developed at a regional oncology center. *Acta Orthop Scand Suppl* **203**, 13–77 (1983).
7. Myhre-Jensen, O. A consecutive 7-year series of 1331 benign soft tissue tumours. Clinicopathologic data. Comparison with sarcomas. *Acta Orthop Scand* **52**, 287–293 (1981).
8. Chung, W. J. *et al*. MRI to differentiate benign from malignant soft-tissue tumours of the extremities: a simplified systematic imaging approach using depth, size and heterogeneity of signal intensity. *Br J Radiol* **85**, e831–836, https://doi.org/10.1259/bjr/27487871 (2012).
9. Calleja, M., Dimigen, M. & Saifuddin, A. MRI of superficial soft tissue masses: analysis of features useful in distinguishing between benign and malignant lesions. *Skeletal Radiol* **41**, 1517–1524, https://doi.org/10.1007/s00256-012-1385-6 (2012).
10. Zhang, Z., Deng, L., Ding, L. & Meng, Q. MR imaging differentiation of malignant soft tissue tumors from peripheral schwannomas with large size and heterogeneous signal intensity. *Eur J Radiol* **84**, 940–946, https://doi.org/10.1016/j.ejrad.2015.02.003 (2015).
11. Berquist, T. H., Ehman, R. L., King, B. F., Hodgman, C. G. & Ilstrup, D. M. Value of MR imaging in differentiating benign from malignant soft-tissue masses: study of 95 lesions. *AJR Am J Roentgenol* **155**, 1251–1255, https://doi.org/10.2214/ajr.155.6.2122675 (1990).
12. Banks, K. P. The target sign: extremity. *Radiology* **234**, 899–900, https://doi.org/10.1148/radiol.2343030946 (2005).
13. Murphy, M., Smith, W., Smith, S., Kransdorf, M. & Temple, H. Imaging of musculoskeletal neurogenic tumors: radiologic-pathologic correlations. *Radiographics* **19**, 1253–1280 (1999).
14. Walker, E. A., Song, A. J. & Murphey, M. D. Magnetic resonance imaging of soft-tissue masses. *Semin Roentgenol* **45**, 277–297, https://doi.org/10.1053/j.ro.2009.12.004 (2010).
15. Lefkowitz, R. A. *et al*. Myxofibrosarcoma: prevalence and diagnostic value of the "tail sign" on magnetic resonance imaging. *Skeletal Radiol* **42**, 809–818, https://doi.org/10.1007/s00256-012-1563-6 (2013).
16. De Schepper, A. M., De Beuckeleer, L., Vandevenne, J. & Somville, J. Magnetic resonance imaging of soft tissue tumors. *Eur Radiol* **10**, 213–223, https://doi.org/10.1007/s003300050037 (2000).
17. Gielen, J. L. *et al*. Accuracy of MRI in characterization of soft tissue tumors and tumor-like lesions. A prospective study in 548 patients. *Eur Radiol* **14**, 2320–2330, https://doi.org/10.1007/s00330-004-2431-0 (2004).
18. Kransdorf, M. J. *et al*. Soft-tissue masses: diagnosis using MR imaging. *American Journal of Roentgenology* **153**, 541–547 (1989).
19. Kransdorf, M. J. & Murphey, M. D. Radiologic evaluation of soft-tissue masses: a current perspective. *AJR Am J Roentgenol* **175**, 575–587, https://doi.org/10.2214/ajr.175.3.1750575 (2000).
20. Crim, J. R., Seeger, L. L., Yao, L., Chandnani, V. & Eckardt, J. J. Diagnosis of soft-tissue masses with MR imaging: can benign masses be differentiated from malignant ones? *Radiology* **185**, 581–586, https://doi.org/10.1148/radiology.185.2.1410377 (1992).
21. Pekcevik, Y., Kahya, M. O. & Kaya, A. Characterization of soft tissue tumors by diffusion-weighted imaging. *Iranian Journal of Radiology* **12** (2015).
22. Degnan, A. J., Chung, C. Y. & Shah, A. J. Quantitative diffusion-weighted magnetic resonance imaging assessment of chemotherapy treatment response of pediatric osteosarcoma and Ewing sarcoma malignant bone tumors. *Clinical imaging* **47**, 9–13 (2018).
23. Oka, K. *et al*. The value of diffusion-weighted imaging for monitoring the chemotherapeutic response of osteosarcoma: a comparison between average apparent diffusion coefficient and minimum apparent diffusion coefficient. *Skeletal radiology* **39**, 141–146 (2010).
24. Jeon, J. Y., Chung, H. W., Lee, M. H., Lee, S. H. & Shin, M. J. Usefulness of diffusion-weighted MR imaging for differentiating between benign and malignant superficial soft tissue tumours and tumour-like lesions. *Br J Radiol* **89**, 20150929, https://doi.org/10.1259/bjr.20150929 (2016).
25. Moulton, J. S. *et al*. MR imaging of soft-tissue masses: diagnostic efficacy and value of distinguishing between benign and malignant lesions. *AJR Am J Roentgenol* **164**, 1191–1199, https://doi.org/10.2214/ajr.164.5.7717231 (1995).
26. Kim, H. S., Kim, J.-H., Yoon, Y. C. & Choe, B. K. Tumor spatial heterogeneity in myxoid-containing soft tissue using texture analysis of diffusion-weighted MRI. *PloS one* **12**, e0181339 (2017).
27. Subhawong, T. K., Jacobs, M. A. & Fayad, L. M. Diffusion-weighted MR imaging for characterizing musculoskeletal lesions. *Radiographics* **34**, 1163–1177 (2014).
28. Subhawong, T. K., Jacobs, M. A. & Fayad, L. M. Insights into quantitative diffusion-weighted MRI for musculoskeletal tumor imaging. *AJR Am J Roentgenol* **203**, 560–572, https://doi.org/10.2214/AJR.13.12165 (2014).
29. Chee, D. W., Peh, W. C. & Shek, T. W. Pictorial essay: imaging of peripheral nerve sheath tumours. *Can Assoc Radiol J* **62**, 176–182, https://doi.org/10.1016/j.carj.2010.04.009 (2011).
30. Cho, Y. J. & Kim, J. Y. Alveolar soft part sarcoma: clinical presentation, treatment and outcome in a series of 19 patients. *Clin Orthop Surg* **6**, 80–86, https://doi.org/10.4055/cios.2014.6.1.80 (2014).
31. Goodlad, J. R., Mentzel, T. & Fletcher, C. D. Low grade fibromyxoid sarcoma: clinicopathological analysis of eleven new cases in support of a distinct entity. *Histopathology* **26**, 229–237 (1995).
32. Sundaram, M., Baran, G., Merenda, G. & McDonald, D. J. Myxoid liposarcoma: magnetic resonance imaging appearances with clinical and histological correlation. *Skeletal Radiol* **19**, 359–362 (1990).
33. Chhabra, A. & Soldatos, T. Soft-tissue lesions: when can we exclude sarcoma? *AJR Am J Roentgenol* **199**, 1345–1357, https://doi.org/10.2214/AJR.12.8719 (2012).
34. Chen, C. K. *et al*. Differentiating benign and malignant soft tissue masses by magnetic resonance imaging: role of tissue component analysis. *J Chin Med Assoc* **72**, 194–201, https://doi.org/10.1016/S1726-4901(09)70053-X (2009).
35. Tung, G. A. & Davis, L. M. The role of magnetic resonance imaging in the evaluation of the soft tissue mass. *Crit Rev Diagn Imaging* **34**, 239–308 (1993).
36. Datir, A. *et al*. MRI of soft-tissue masses: the relationship between lesion size, depth, and diagnosis. *Clinical radiology* **63**, 373–378 (2008).

Author Contributions

J.H.L. collected data and wrote the manuscript. Y.C.Y. conceived, designed and supervised the study. Y.C.Y., W.J., J.G.C. analyzed data and interpreted the results. S.K. supervised the study and conducted all statistical analyses. All authors reviewed and approved the manuscript.

Additional Information

Supplementary information accompanies this paper at https://doi.org/10.1038/s41598-019-41230-0.

Competing Interests: The authors declare no competing interests.

Publisher's note: Springer Nature remains neutral with regard to jurisdictional claims in published maps and institutional affiliations.

Open Access This article is licensed under a Creative Commons Attribution 4.0 International License, which permits use, sharing, adaptation, distribution and reproduction in any medium or format, as long as you give appropriate credit to the original author(s) and the source, provide a link to the Creative Commons license, and indicate if changes were made. The images or other third party material in this article are included in the article's Creative Commons license, unless indicated otherwise in a credit line to the material. If material is not included in the article's Creative Commons license and your intended use is not permitted by statutory regulation or exceeds the permitted use, you will need to obtain permission directly from the copyright holder. To view a copy of this license, visit http://creativecommons.org/licenses/by/4.0/.

© The Author(s) 2019
